# Molecular phylogenetics of slit‐faced bats (Chiroptera: Nycteridae) reveal deeply divergent African lineages

**DOI:** 10.1111/jzs.12313

**Published:** 2019-08-20

**Authors:** Terrence C. Demos, Paul W. Webala, Julian C. Kerbis Peterhans, Steven M. Goodman, Michael Bartonjo, Bruce D. Patterson

**Affiliations:** ^1^ Integrative Research Center, Field Museum of Natural History Chicago Illinois; ^2^ Department of Forestry and Wildlife Management Maasai Mara University Narok Kenya; ^3^ College of Arts and Sciences Roosevelt University Chicago Illinois; ^4^ Association Vahatra Antananarivo Madagascar; ^5^ Mammalogy Section National Museums of Kenya Nairobi Kenya

**Keywords:** Africa, biodiversity, *Nycteris*, species tree, taxonomy

## Abstract

The bat family Nycteridae contains only the genus *Nycteris*, which comprises 13 currently recognized species from Africa and the Arabian Peninsula, one species from Madagascar, and two species restricted to Malaysia and Indonesia in South‐East Asia. We investigated genetic variation, clade membership, and phylogenetic relationships in Nycteridae with broad sampling across Africa for most clades. We sequenced mitochondrial cytochrome *b* (*cytb*) and four independent nuclear introns (2,166 bp) from 253 individuals. Although our samples did not include all recognized species, we recovered at least 16 deeply divergent monophyletic lineages using independent mitochondrial and multilocus nuclear datasets in both gene tree and species tree analyses. Mean pairwise uncorrected genetic distances among species‐ranked *Nycteris* clades (17% for *cytb* and 4% for concatenated introns) suggest high levels of phylogenetic diversity in Nycteridae. We found a large number of designated clades whose members are distributed wholly or partly in East Africa (10 of 16 clades), indicating that *Nycteris* diversity has been historically underestimated and raising the possibility that additional unsampled and/or undescribed *Nycteris* species occur in more poorly sampled Central and West Africa. Well‐resolved mitochondrial, concatenated nuclear, and species trees strongly supported African ancestry for SE Asian species. Species tree analyses strongly support two deeply diverged subclades that have not previously been recognized, and these clades may warrant recognition as subgenera. Our analyses also strongly support four traditionally recognized species groups of *Nycteris*. Mitonuclear discordance regarding geographic population structure in *Nycteris thebaica* appears to result from male‐biased dispersal in this species. Our analyses, almost wholly based on museum voucher specimens, serve to identify species‐rank clades that can be tested with independent datasets, such as morphology, vocalizations, distributions, and ectoparasites. Our analyses highlight the need for a comprehensive revision of Nycteridae.

## Graphical abstract

We investigated genetic variation, clade membership, and phylogenetic relationships in the bat family Nycteridae with broad sampling across Africa. At least 16 deeply divergent monophyletic lineages were recovered in both gene tree and species tree analyses. Genetic distances among species‐ranked *Nycteris* clades (17% for *cytb *and 4% for concatenated introns) suggest high levels of phylogenetic diversity in Nycteridae. A large number of clades are distributed wholly or partly in East Africa (10/16 clades), suggesting *Nycteris* diversity has been historically underestimated.

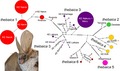

## 1 INTRODUCTION

The Paleotropical slit‐faced bats, family Nycteridae, all belong to the genus *Nycteris* with 13 of 16 recognized species found in continental Africa and offshore islands, one species on Madagascar, and two species endemic to South‐East Asia (Mammal Diversity Database, 2019; Simmons, 2005). Members of the Nycteridae are readily recognizable by their nose leaves, which are divided by a deep median furrow running the length of the muzzle, the basis for their common name. They also possess a Y‐shaped terminal caudal vertebra that is unique among mammals. Systematic reviews of the family have not been informed by morphological or molecular phylogenetics, and the most recently named species in the family was described a half‐century ago (*N. vinsoni*, Dalquest, 1965). To put this taxonomic stasis in context, the number of recognized bat species globally has grown by 26.4% over the last 15 years. In the Paleotropics, this has included a 38% increase in the number of species of Rhinolophidae and a >50% increase in species in the genera *Scotophilus* and *Miniopterus* (cf. Simmons, 2005; Mammal Diversity Database, 2019). Here, we use a geographically extensive, multilocus dataset to assay the diversity and infer the evolutionary relationships of Nycteridae in order to establish the foundations for a fuller taxonomic revision.

**Figure 1:**
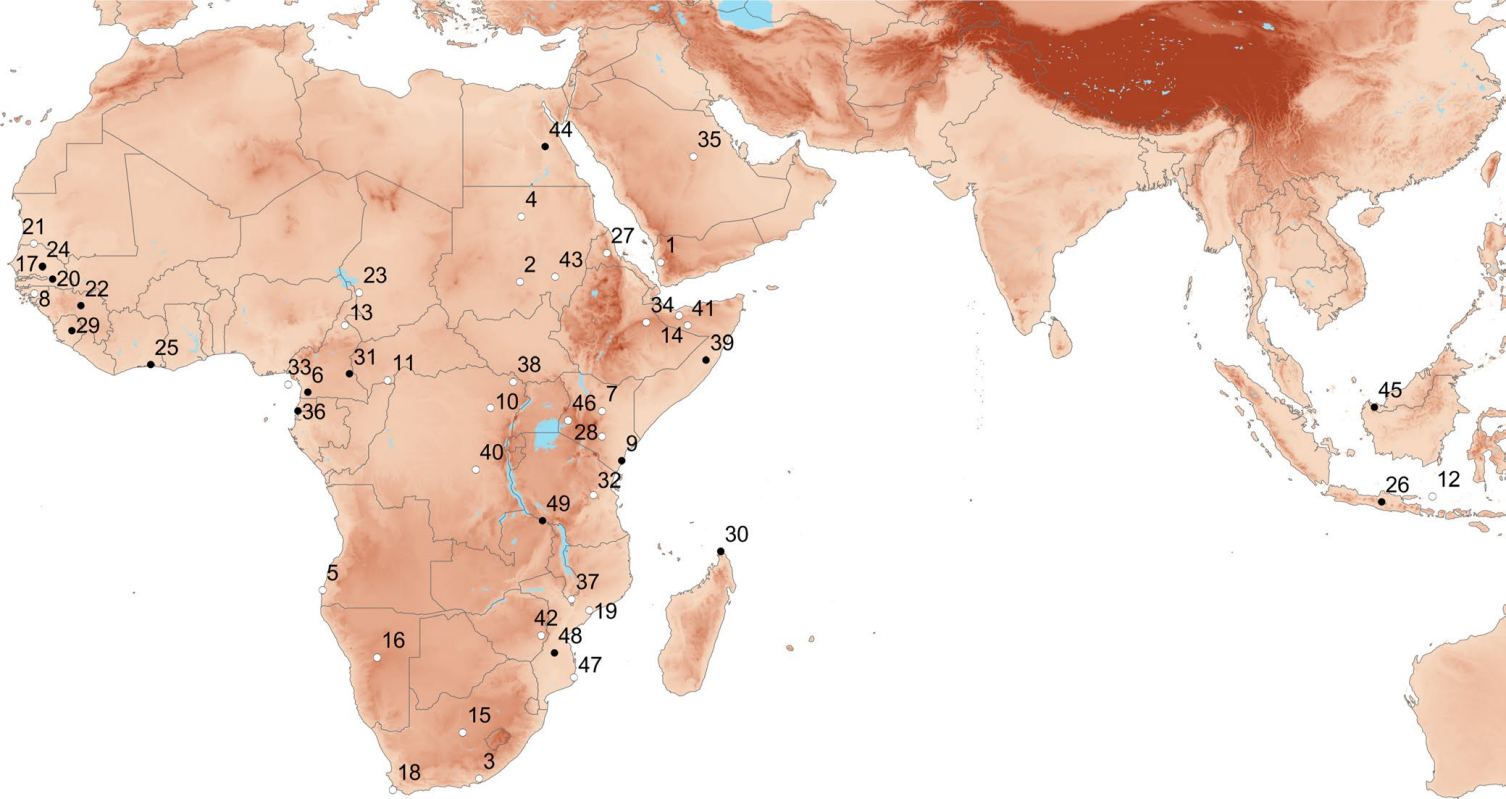
Named taxa of *Nycteris*, showing type localities for recognized species (filled circles) and subspecies or synonyms (open circles). Number codes are as follows: 1 – *adana* K. Andersen, 1912; 2 – *aethiopica* Dobson, 1878; 3 – *affinis* A. Smith, 1829; 4 –*albiventer* Wagner, 1840; 5 – *angolensis* Peters, 1871; 6 – *arge* Thomas, 1903; 7 – *aurantiaca* De Beaux, 1923; 8 – *aurantiaca* Monard, 1939; 9 – *aurita* K. Andersen, 1912; 10 – *avakubia* J. A. Allen, 1917; 11 – *baikii* Gray 1867; 12 – *bastiani* Bergmans & van Bree, 1986; 13 – *benuensis* Aellen, 1952; 14 – *brockmani* K. Andersen, 1912; 15 – *capensis* A. Smith, 1829; 16 – *damarensis* Peters, 1871; 17 – *daubentonii*. Geoffroy, 1813; 18 – *discolor* Wagner, 1840; 19 – *fuliginosa* Peters, 1852; 20 – *gambiensis* K. Andersen, 1912; 21 – *geoffroyi* Desmarest, 1820; 22 – *grandis* Peters, 1865; 23 – *guineensis* Monard, 1939; 24 – *hispida* Schreber, 1775; 25 – *intermedia* Aellen, 1959; 26 – *javanica*. Geoffroy, 1813; 27 – *labiata* Heuglin, 1861; 28 – *luteola* Thomas, 1901; 29 – *macrotis* Dobson, 1876; 30 – *madagascariensis* G. Grandidier, 1937; 31 – *major* K. Andersen, 1912; 32 – *marica* Kershaw, 1923; 33 – *martini* Fraser, 1843; 34 – *media* K. Andersen, 1912; 35 – *najdiya* Nader & Kock, 1982; 36 – *nana* K. Andersen, 1912; 37 – *oriana* Kershaw, 1922; 38 – *pallida* J. A. Allen, 1917; 39 – *parisii* De Beaux, 1924; 40 – *proxima* Lonnberg & Gyldenstolpe, 1925; 41 – *revoilii* Robin, 1881; 42 – *sabiensis* Roberts, 1946; 43 – *senegalensis* Hartmann, 1868; 44 – *thebaica*. Geoffroy, 1818; 45 – *tragata* K. Andersen, 1912; 46 – *tristis* G. M. Allen & Lawrence, 1936; 47 – *villosa* Peters, 1852; 48 – *vinsoni* Dalquest, 1965; and 49 – *woodi* K. Andersen, 1914. An additional name, *pilosa* Gray, 1866 from “Africa,” is not shown

In the first systematic revision of Nycteridae, Andersen (1912) divided then‐known taxa into four species groups: *javanica, hispida, aethiopica* [now known as *macrotis*], and *thebaica*. Later, Aellen (1959) divided the *javanica* group into two based on tragus and dental characters: *javanica* (monotypic) and *arge*, which contained both African and Asian species. Using morphometrics and hyoid morphology, respectively, Van Cakenberghe and De Vree (1993a) and Griffiths (1997) later transferred the Asian member of the *arge* group*, N. tragata*, to the *javanica* group. This five‐group classification has been widely accepted (e.g., Simmons, 2005), but taxonomic membership in these groups has varied, owing to mosaic character variation. For example, the absence of biometrical differences in teeth measurements suggested the conspecificity of *N. parisii* with *N. woodi* (Van Cakenberghe & de Vree, 1985), but a subsequent study of bacula strongly supported the validity of both species and suggested their assignment to entirely different species groups (Thomas, Harrison, & Bates, 1994). Although qualitative and mensural characters have been used to characterize and differentiate species, external and skull characters are in conflict with other morphological characters (e.g., Happold, 2013a; Monadjem, Taylor, Cotterill, & Schoeman, 2010; Thomas et al., 1994; Van Cakenberghe & de Vree, 1985, 1993a, 1993b, 1998). Except for Griffiths’ (1997) analysis of the hyoid apparatus, the morphological characters of the species of Nycteridae have not been subjected to explicit phylogenetic analysis. Figure 1 shows the host of names available for *Nycteris* populations, many of them currently considered synonyms (cf. Simmons, 2005).

Molecular phylogenetic analyses of the Nycteridae are likewise limited, as they included only a handful of species, each represented by a single sample. Shi and Rabosky (2015) used a concatenated supermatrix and included 7 of 16 *Nycteris* species in a time‐calibrated analysis of all Chiroptera. They found strong support for the traditional sister relationship between Nycteridae and Emballonuridae (the two families comprising the Emballonuridea of Koopman, 1993). The supermatrix analysis of Amador, Moyers Arévalo, Almeida, Catalano, and Giannini (2018), also based on the same seven *Nycteris* species, found inconsistent evidence for the endemic Malagasy Myzopodidae joining this group. Nevertheless, both studies recovered Nycteridae as monophyletic and a close relative of Emballonuridae, and both studies recovered the two Asian species, *N. tragata* and *N. javanica*, as well‐supported sisters. It should be noted, however, that both studies were based on incomplete supermatrices (71% missing data in Amador et al., 2018 and 83% missing in Shi & Rabosky, 2015). Thus, the diversity and phylogenetic relationships of species in Nycteridae remain largely unresolved and the evolutionary independence of *Nycteris* lineages has yet to be established.

Bat surveys across Africa over the last two decades have provided substantial new material for the evaluation of phylogenetic relationships and species limits. In addition, recent studies (Demos, Webala, Bartonjo, & Patterson, 2018; Dool et al., 2016; Patterson et al., 2018) have shown that a multilocus intron system based on different chromosomes and enabling independent representation of the nuclear genome offers clear advantages over analyses based only on mitochondrial data. Advantages include better resolution of earlier divergences (e.g., Demos et al., 2019) and improved detection of instances of mitochondrial introgression (e.g., Dool et al., 2016; Hassanin et al., 2018). Here, we address three key aspects of Nycteridae evolution: (a) recognizing monophyletic lineages within *Nycteris*, focusing on Afrotropical species, and assessing their evolutionary independence using independent nuclear loci under a coalescent framework; (b) evaluating their phylogenetic relationships using both nuclear and mitochondrial data in gene tree, concatenated, and species tree analyses; and (c) assessing the species‐group relationships of *Nycteris* species that had been classified by morphology alone. This study highlights the need for a comprehensive revision of African Nycteridae. Our analyses and discussion serve to identify species‐rank clades that need to be tested with independent datasets including morphology, vocalizations, distributions, and ectoparasites.

## 2 MATERIALS AND METHODS

### 2.1 Selection of taxa and sampling

The bats newly sequenced for this study (*n* = 249) were collected during recent small mammal surveys across sub‐Saharan Africa, with relatively dense sampling in East Africa (see Figure S1 in Supporting Information). Initial assignment of individuals to species for East African specimens was determined using meristic, mensural, and qualitative characters presented in the bat keys of Thorn, Kerbis Peterhans, and Baranga (2009) and Patterson and Webala (2012). Field methods followed mammal collecting guidelines (Sikes, 2016) and were approved under Field Museum of Natural History IACUC #2012‐003. Tissues were taken from euthanized specimens in the course of preparing voucher specimens following IACUC protocols and the respective national collecting permits. Tissues were variously preserved in ethanol, saturated salt solution (EDTA‐DMSO‐NaCl), or liquid nitrogen and stored in liquid nitrogen dewars. Four additional *cytochrome b gene* (*cytb*) sequences of *Nycteris* were downloaded from GenBank. *Coleura afra* (Emballonuridae) was included as an out‐group. In total, 1–5 genes were analyzed in 253 individuals in this study (see Table S1 in Supporting Information for voucher numbers and locality data and Appendix 1 for GenBank accession numbers). To enable subsequent integrative taxonomic revisions, all but four of the individuals analyzed genetically in this study are accompanied by museum voucher specimens suitable for morphological analysis.

In view of the large number of names (many of which are synonyms; Figure 1) and to avoid contributing to current taxonomic confusion in *Nycteris*, we utilized a conservative approach in labeling clades. Where a clade's taxonomic identity was ambiguous or unknown, we referred to it simply as a numbered clade. In some cases, even assignment to equivocal groupings was necessary (e.g., *hispida/aurita* and cf. *hispida/aurita*). Although used as explicit labels in our study, the validity of these names is provisional. Comprehensive morphological assessments of individual specimens making up these clades included in our analyses will be required in order to verify which, if any, existing names may apply to them.

### 2.2 Amplification and sequencing

We sequenced one mitochondrial protein‐coding gene *cytochrome b* (*cytb*) and the nuclear introns *acyl‐CoA oxidase 2 intron 3* (*ACOX2*), *COP9 signalosome subunit 7A intron 4* (*COPS7A*), *rogdi atypical leucine zipper intron 7* (*ROGDI*), and *signal transducer and activator of transcription 5A intron* (*STAT5A*) for specimens of *Nycteris* and the close emballonurid out‐group *Coleura afra*. Primers, primer references, and thermocycler conditions are described in Table 1. General methods of DNA extraction, amplification, and sequencing follow Demos et al. (2018) and Patterson et al. (2018). DNA sequences were assembled, aligned, and edited using GENEIOUS PRO v.11.1.5 (Biomatters Ltd.). Alignments were inspected visually and determined to be unambiguous. Several gaps were introduced in the alignments of the four nuclear introns, but their positions were unambiguous. Sequences of *cytb* were translated to amino acids to confirm the absence of premature stop codons and indels. The *cytb* alignment was trimmed to 1,121 nucleotides to minimize missing data. Before phylogenetic analyses using mitochondrial data, we reduced the matrix of 253 individuals to the set of unique sequences, resulting in a final matrix of 164 individuals. The matrix used for calculating *cytb* distances between lineages comprised 250 individuals from the 253 individual alignments. We resolved nuclear DNA to haplotypes with the PHASE program (Stephens, Smith, & Donnelly, 2001) and set the probability threshold to 70%, following Garrick, Sunnucks, and Dyer (2010). PHASE files were formatted and assembled using SeqPhase (Flot, 2010).

**Table 1:** Primer information for genes amplified in the current study. References indicated by (a) Salicini, Ibáñez, & Juste, 2011; (b) Eick, Jacobs, & Matthee, 2005; (c) Trujillo, Patton, Schlitter, & Bickham, 2009)

Gene	Primers (5’–3’)	Amplicon length	References	Thermal profile
*ACOX2*	ACOX2f CCTSGGCTCDGAGGAGCAGAT ACOX2r GGGCTGTGHAYCACAAACTCCT	717 bp	a	3 min at 95°C followed by 10 cycles of 15 s at 95°C, 30 s at 65°C in 1°C decrements from 65°C (64–56°C), and 1 min at 72°C, followed by 36 cycles of 15 s at 95°C, 30 s at 55°C, and 1 min at 72°C, and final 5 min extension at 70°C
*COPS7A*	COPSf TACAGCATYGGRCGRGACATCCA COPSr TCACYTGCTCCTCRATGCCKGACA	689 bp	a	Same as *ACOX2* above
*ROGDI*	ROGDIf CTGATGGAYGCYGTGATGCTGCA ROGDIr CACGGTGAGGCASAGCTTGTTGA	505 bp	a	3 min at 95°C followed by 10 cycles of 15 s at 95°C, 30 s at 60°C in 1°C decrements from 60°C (59–51°C), and 1 min at 72°C, followed by 36 cycles of 15 s at 95°C, 30 s at 50°C, and 1 min at 72°C, and final 5 min extension at 70°C
*STAT5A*	STAT5f CTGCTCATCAACAAGCCCGA STAT5r GGCTTCAGGTTCCACAGGTTGC	530 bp	b	Same as *ROGDI* above
*cytb*	LGL−765f GAAAAACCAYCGTTGTWATTCAACT LGL−766r GTTTAATTAGAATYTYAGCTTTGGG		c	3 min at 95°C followed by 36 cycles of 45 s at 95°C, 30 s at 50°C, and 2.5 min at 70°C, and final 5 min extension at 70°C

### 2.3 Gene trees, networks, species trees, and summary statistics

PartitionFinder 2 (Lanfear, Frandsen, Wright, Senfeld, & Calcott, 2016) on CIPRES Science Gateway v.3.1 (Miller, Pfeiffer, & Schwartz, 2010) was used to determine the appropriate model of sequence evolution using the Bayesian information criterion (BIC) for *cytb* and the four nuclear introns. Interspecific uncorrected sequence divergences (*p‐*distances) for *cytb* were calculated for both positions 1, 2, and 3 and positions 1 and 2 only, and intraspecific distances were calculated using positions 1, 2, and 3 using MEGA X 10.0.5 (Kumar, Stecher, Li, Knyaz, & Tamura, 2018).

Maximum‐likelihood (ML) inference of *cytb* gene trees and a concatenated alignment using four partitioned nuclear introns were made using the program IQ‐TREE version 1.6.0 (Nguyen, Schmidt, von Haeseler, & Minh, 2015) on the CIPRES portal. Gene tree analyses under a Bayesian inference (BI) framework were carried out in MRBAYES v.3.2.6 (Ronquist et al., 2012) on the CIPRES portal to infer gene trees for *cytb* and the partitioned alignment of four nuclear introns. Two replicates were run in MrBayes, and nucleotide substitution models were unlinked across partitions for each nuclear locus in the concatenated alignment. Four Markov chains were run for 1 × 10^7^ generations using default heating values and sampled every 1000th generation. Stationarity of the MRBAYES results was assessed in Tracer v1.7 (Rambaut, Drummond, Xie, Baele, & Suchard, 2018). Majority‐rule consensus trees were inferred for each Bayesian analysis. PopART (Leigh & Bryant, 2015) was used to construct a median‐joining network of cytochrome *b* haplotypes for clades within *Nycteris thebaica.* Pie charts were used to visualize the relative frequencies and relationships of haplotypes in *N. thebaica* clades 1–6.

*Nycteris* taxa were assigned to either species or named clades based on clade support in the analyses of the *cytb* and nuclear intron datasets. As in Demos et al. (2018), results from gene tree analyses were used to identify populations to be used as “candidate species” for the species tree approach implemented in StarBEAST2 (Ogilvie, Bouckaert, & Drummond, 2017), an extension of BEAST v.2.5.1 (Bouckaert et al., 2014). Species tree analyses were carried out using the four nuclear intron alignments with substitution, clock, and tree models unlinked among loci. The lognormal relaxed‐clock model was applied to each locus using a Yule tree prior and the linear with constant root population size model. Four replicates were carried out, and the analyses were run for 2 × 10^8^ generations with 10% of each run discarded as burn‐in. We used Tracer v.1.7 to assess convergence and stationarity of model parameters based on ESS values and examination of trace files.

Sequence alignments used in this study have been deposited on the Figshare data repository (https://doi.org/10.6084/m9.figshare.8081594.v1). All newly generated sequences are available on GenBank with accession numbers MK837076–MK837603 (see also Appendix 1).

## 3 RESULTS

### 3.1 Mitochondrial genetic diversity, gene trees, and haplotype network

Sequences were generated and aligned for *cytb* (1,121 bp, 99% coverage), *ACOX2* (646 bp, 96% coverage), *COPS7A* (624 bp, 98% coverage), *ROGDI* (450 bp, 98% coverage), and *STAT5A* (523 bp, 98% coverage). The concatenated alignment of four introns for 70 individuals was 97.1% complete (mean sequence length 2,166 bp). Models of sequence evolution inferred by PartitionFinder 2 were as follows: *cytb*, GTR + I+G; *ACOX2*, TrN + G; *COPS7A*, TrN + G; *ROGDI*, TrN + G; and *STAT5A*, TrN + G. Uncorrected *cytb* distances for reciprocally monophyletic *Nycteris* lineages in the 250 sequence *cytb* alignment ranged from 3.6% to 22.2% for *cytb* positions 1 + 2 + 3 and 1.0%–8.0% for *cytb* positions 1 + 2 (Table 2). Within‐lineage variability for *cytb* positions 1 + 2 + 3 ranged from 0% to 4.9%.

**Table 2:** Uncorrected *cytb p*‐distances among clades of *Nycteris*: on and below diagonal based on positions 1, 2, and 3; above diagonal, positions 1 and 2. Clades represented by one individual (*N.* cf. *thebaica* 3, *N. javanica*, *N. nana* 1) not included

	Taxon	[1]	[2]	[3]	[4]	[5]	[6]	[7]	[8]	[9]	[10]	[11]	[12]	[13]	[14]	[15]	[16]	[17]	[18]
[1]	*arge* 1	**4.3**	3.7	3.8	7.6	5.3	3.7	3.6	4.2	4.1	4.9	4.0	6.5	6.6	6.4	6.4	6.5	6.6	3.1
[2]	*arge* 2	15.9	**1.1**	4.5	7.3	6.3	3.7	3.4	4.5	3.6	4.6	2.6	6.4	6.7	6.5	6.7	6.7	6.5	3.6
[3]	cf. *hispida/aurita*	16.1	17.5	**0.7**	8.0	6.4	3.9	4.0	5.3	4.3	5.3	4.6	7.5	7.4	7.0	7.0	7.5	7.0	4.1
[4]	cf. *thebaica* 1	19.3	19.2	19.5	**2.6**	6.2	7.4	7.7	8.0	7.1	6.9	7.9	6.7	7.2	7.3	6.8	7.3	7.0	6.0
[5]	cf. *thebaica* 2	17.0	17.8	19.0	14.9	**0.1**	6.0	6.3	5.8	5.5	6.3	6.1	5.9	6.1	6.0	5.7	6.0	5.8	6.0
[6]	*grandis*	16.3	16.3	17.2	20.3	18.2	**1.6**	3.7	3.9	4.2	4.9	4.4	7.1	7.2	7.2	7.2	7.0	7.1	3.6
[7]	*hispida/aurita*	14.5	15.4	15.0	19.7	18.0	16.1	**2.5**	5.0	4.5	4.8	4.0	6.9	7.4	7.2	7.0	7.5	7.5	3.2
[8]	*macrotis* 1	17.4	18.1	18.1	20.8	19.5	17.7	17.7	**2.2**	3.3	4.0	4.9	6.9	7.1	6.9	7.1	6.6	6.8	3.9
[9]	*macrotis* 2	16.3	18.7	17.8	19.4	19.0	18.5	16.3	13.8	**0.9**	3.7	4.1	7.8	7.5	7.2	7.2	7.1	7.1	3.7
[10]	*macrotis* 3	17.6	19.0	19.2	20.1	20.0	18.7	17.8	14.3	15.0	**0.4**	5.0	7.4	7.8	8.0	7.7	7.6	7.7	4.4
[11]	*nana* 2	16.1	13.2	17.1	19.3	16.8	17.0	15.3	17.8	17.4	18.0	**4.9**	6.9	7.1	7.0	7.0	6.9	7.0	4.4
[12]	*thebaica* 1	18.7	18.5	19.1	18.1	17.1	19.4	19.0	19.6	22.2	20.1	18.0	**0.4**	2.0	1.9	2.0	2.5	1.9	6.4
[13]	*thebaica* 2	18.7	18.4	19.9	18.4	17.8	19.7	19.4	20.1	21.5	20.4	18.2	5.8	**0.4**	1.3	1.7	2.3	1.4	7.3
[14]	*thebaica* 3	18.9	18.6	19.5	18.2	18.0	19.9	19.6	20.4	21.8	21.1	18.5	5.0	5.0	**1.6**	1.2	1.6	1.0	7.1
[15]	*thebaica* 4	18.4	18.6	19.7	17.7	17.0	19.9	19.5	19.9	21.6	20.4	17.9	5.1	4.7	3.6	**1.6**	2.3	1.3	6.9
[16]	*thebaica* 5	18.9	19.2	19.7	18.6	17.5	19.5	20.0	19.6	21.9	20.4	18.4	6.4	6.5	5.6	5.3	**0.0**	1.4	7.3
[17]	*thebaica* 6	18.2	18.1	19.5	17.4	17.0	18.8	19.4	19.8	21.4	19.7	17.4	5.4	5.2	4.7	4.2	5.4	**1.7**	7.3
[18]	*tragata*	14.4	17.7	17.2	18.6	18.7	15.8	16.3	18.2	17.9	16.3	17.0	17.0	18.1	17.9	17.9	18.3	17.2	**1.3**

**Figure 2:**
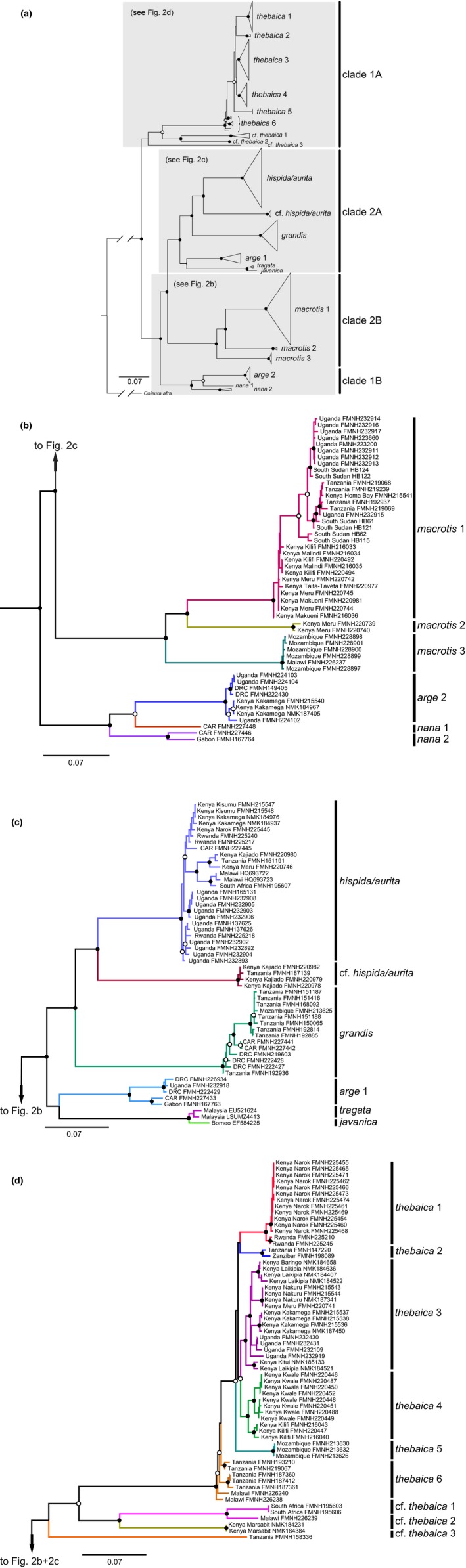
(a) Maximum‐likelihood phylogeny of 163 *Nycteris* specimens based on cytochrome *b*. The phylogeny was inferred in IQ‐TREE and its topology closely resembled the phylogeny calculated in MrBayes under a Bayesian framework. Filled circles on nodes denote bootstrap values (BS) ≥70% and Bayesian posterior probabilities (PP) ≥0.95, open circles outlined in black indicate BS ≥ 70% and PP < 0.95, and unmarked nodes indicate BS < 70% and PP < 0.95. Support values for most minor clades are not shown. Species names assigned on basis of preliminary field identifications or examination of museum specimens. (b–d) enlarged sections of the complete *cytb* tree showing individual relationships. Specimen localities include counties for densely sampled Kenya. CAR refers to Central African Republic and DRC to Democratic Republic of the Congo. Museum acronyms are defined in Appendix 1

The ML phylogeny for Nycteridae based on *cytb* shows division of the family into four deeply diverged subclades (labeled as clades 1A, 1B, 2A, and 2B in Figure 2a). The topology of the maximum clade credibility tree is substantially similar in topology to the maximum‐likelihood tree presented here. The monophyly of all named clades was strongly supported with the exception of *Nycteris thebaica* clade 6. Relationships among clades were generally well supported with the exception of the position of (a) the relationships of the geographically delimited clades within *N. thebaica*, (b) *N*. cf. *thebaica* clade 3, and (c) the relationship of *N. arge* clade 1 and *N. tragata* + *N. javanica*. Two nodes had equivocal support (bootstrap (BS) ≥70%, posterior probability (PP) <0.95): the node uniting *N. thebaica* clades 1–6 and *N.* cf. *thebaica* clades 1 + 2 and the node uniting *N. arge* clade 2 and *N. nana* clade 1. Several clades with broad geographic sampling showed relatively high levels of within‐clade genetic variation (i.e., *N. hispida*/*aurita*, *N. grandis*, and *N. macrotis* clade 1). For those clades with limited geographic sampling, we recovered high levels of divergence among populations in *N.* cf. *thebaica* 1 and *N. nana* clade 2. Both ML and BI analyses strongly supported *N. arge* clade 1 (Central African Republic [CAR], Democratic Republic of Congo [DRC], Gabon, Uganda) + *N. tragata* (Malaysia) + *N. javanica* (Borneo) as nested well within the other African *Nycteris* clades. The ML and BI trees support multiple deeply divergent clades separated by >10% *cytb* distances. The number of deeply diverged clades that include individuals from East Africa (Kenya, Tanzania, and Uganda) is high: 10 of 16 clades in the trees include individuals from this region.

**Figure 3:**
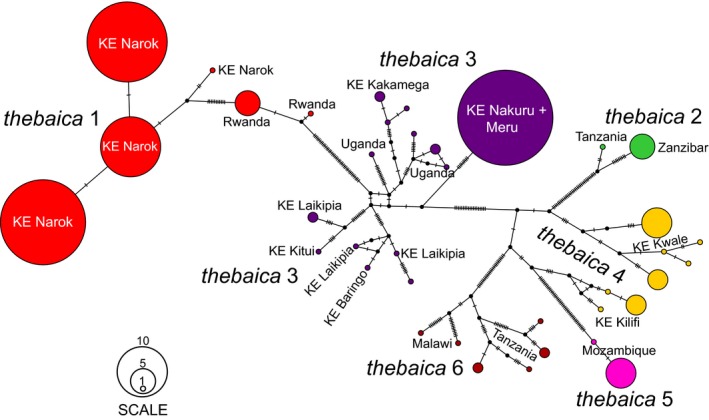
PopART network median‐joining analysis of cytochrome *b* haplotypes for 127 individuals representing *Nycteris thebaica* clades 1 to 6. Colored circles represent different sampled haplotypes, and black circles represent inferred missing or unsampled states. Hatch marks each denote a mutational step between haplotypes. CAR refers to Central African Republic, DRC to Democratic Republic of the Congo, and KE to Kenya

The median‐joining network of *cytb* haplotype diversity for the six allopatric populations within *N. thebaica* showed no shared alleles among clades (Figure 3). The haplotype network revealed the existence of six well‐differentiated clades (minimum separation of clades was 19 substitutions), although *N. thebaica* clade 4 (coastal Kenya) clusters ambiguously between *N. thebaica* clade 5 (Mozambique) and *N. thebaica* clade 2 (Tanzania and Zanzibar).

### 3.2 Concatenated nuclear gene trees

**Figure 4:**
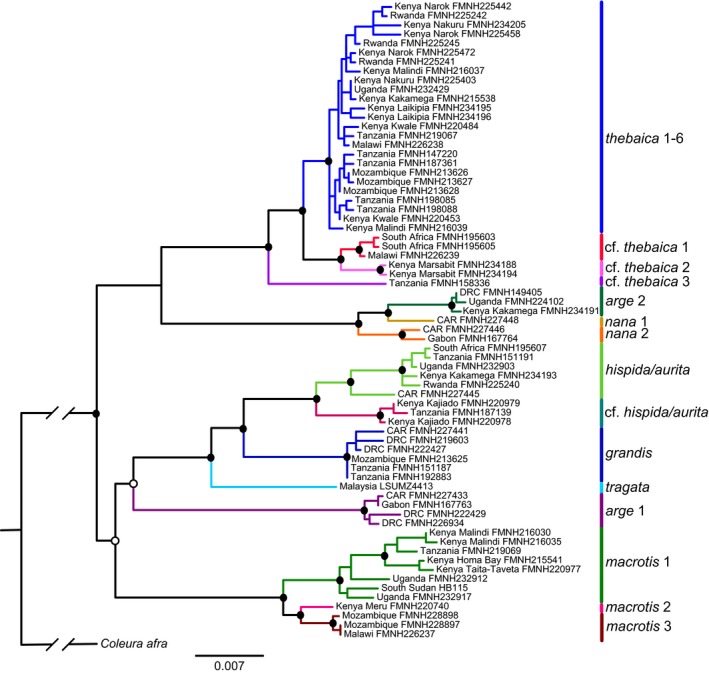
Concatenated Bayesian phylogeny of four independent nuclear introns of *Nycteris.* Filled circles at nodes denote ML bootstrap values (BS) ≥70% and Bayesian posterior probabilities (PP) ≥0.95, open circles outlined in black indicate BS ≥ 70% and PP < 0.95, and unmarked nodes indicate BS < 70% and PP < 0.95. Support values for most minor clades are not shown. Specimen localities include counties for Kenya. CAR refers to Central African Republic and DRC to Democratic Republic of the Congo. Museum acronyms are defined in Appendix 1

The ML gene tree inferred from the concatenated nuclear genes *ACOX2*, *COPS7A*, *ROGDI*, and *STAT5A* (70 individuals; matrix > 97% complete) is shown in Figure 4. This tree was similar to the BI tree with strong support for 22 of 25 major nodes. All of the named clades are strongly supported as monophyletic. Unlike the *cytb* gene trees, the position of *N. arge* clade 2 + *N. nana* clade 1 + *N. nana* clade 2 is ambiguous, while *N.* cf. *thebaica* clade 3 is strongly supported as part of the *N. thebaica* group. *Nycteris tragata* from SE Asia is strongly supported as nested within African *Nycteris* clades but is not sister to *N. arge* clade 1 as in the *cytb* gene trees. The most striking difference between the concatenated nuclear trees and the mitochondrial gene trees is the absence of support for genetic structure among the numbered lineages of *N. thebaica*. None of the clades named as *N. thebaica* 1–6 are supported as monophyletic, and relationships among individuals are poorly supported.

### 3.3 Species trees

**Figure 5:**
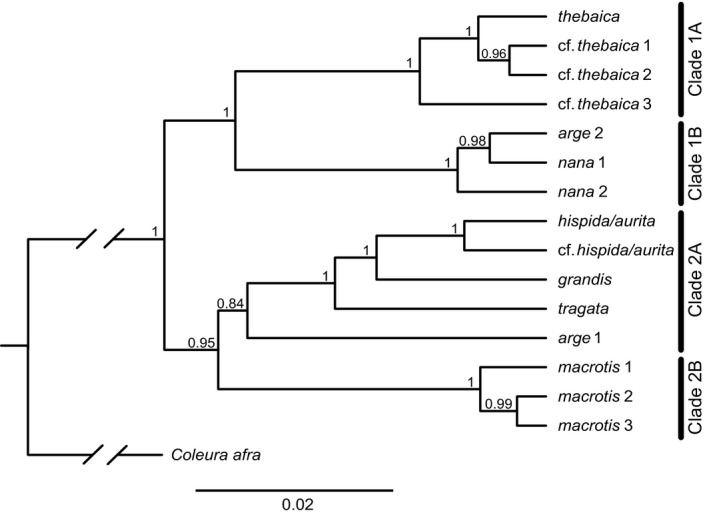
Species tree for *Nycteris* inferred using four nuclear loci in StarBEAST. Nodes are labeled with posterior probabilities

Samples from parameter values of the four StarBEAST analyses had ESS values >200, with the exception of the five tree‐height parameters which all had values >100. We discarded the first 10% of each run, leaving 18,000 species trees in the posterior distributions that were then merged using LogCombiner. The topology of the maximum clade credibility tree (Figure 5) was identical across all four replicates. Species tree analysis using StarBEAST resulted in a topology that is strongly supported, with 12 of 13 nodes having PP ≥ 0.95. As in the concatenated nuclear gene trees, but unlike the *cytb* gene trees, *Nycteris* cf. *thebaica* 3 is strongly supported as sister to the other *N. thebaica* clades. There is strong support for the node uniting *N. arge* 2 + *N. nana* 1 + *N. nana* 2 with the *N. thebaica* clades, resolving a relationship that was poorly supported in all of the gene tree analyses. Most relationships among *N. thebaica* clades 1–6 are poorly supported and minimally diverged, consistent with the assignment of individuals from all six clades to *N. thebaica* (Supporting Information Figure S1). *N. arge* 1 is weakly supported as sister to the strongly supported grouping *N. hispida*/*aurita* + *N*. cf. *hispida*/*aurita* + *N. grandis* + *N. tragata*. *Nycteris tragata*, the only Asian species tested, is well supported within the African clades.

## 4 DISCUSSION

### 4.1 Multiple deeply diverged lineages

The monogeneric Nycteridae has been estimated to have diverged from Emballonuridae 51–53 Mya (Amador et al., 2018; Shi & Rabosky, 2015), and the most recent common ancestor age for the family has been placed variously at 18 mya (Shi & Rabosky, 2015) to 33.9 mya (Amador et al., 2018); Nycteridae ranks as a relatively ancient lineage among Chiroptera. Ours is the most taxonomically and geographically comprehensive phylogenetic study of Nycteridae to date. We recovered multiple instances of deep lineage divergence at both the inter‐ and intra‐clade levels. Mean pairwise uncorrected genetic distances among species‐ranked *Nycteris* clades for *cytb* were 0.17. In comparison, and in equivalent systematic surveys, overall *cytb* distances in *Scotophilus* (0.10; Demos et al., 2018) and *Rhinolophus* (0.10; Demos et al., 2019) were less than that of *Nycteris*. Overall mean genetic distances for concatenated intron datasets showed parallel variation: The mean distance of *Nycteris* was 0.04, *Rhinolophus* was 0.02, and *Scotophilus* was 0.01. As elaborated below, two deeply diverged multispecies clades are apparent in all of the phylogenetic analyses that we executed.

One of the most striking contrasts between the *cytb* gene tree (Figure 2d) and both the concatenated nuclear tree and species tree (Figure 4 and Supporting Information Figure S2) is the pattern of fine‐scale geographic structure for *N. thebaica* apparent only in the mitochondrial tree: There is strong support for monophyly of 5 of 6 labeled *N. thebaica* clades. Population‐level sampling recovered well‐supported and geographically restricted clades in (1) Kenya + Rwanda, (2) Tanzania. (3) Kenya + Uganda, (4) Kenya, and (5) Mozambique (Figure 3). The most divergent of these clades, *N. thebaica* clade 5 from Mozambique, is >5% *cytb* diverged from sister *N. thebaica* clades (Figure 2a, d). However, little population structure is present in either the concatenated nuclear analyses (Figure 4) or in the alternate species tree analysis where individuals were assigned to “species” based on clade membership in the mitochondrial tree (Supporting Information Figure S2). Although incomplete lineage sorting may be expected to play a role in mitonuclear discordance at this phylogenetic level, we note that other haplogroups did not exhibit such discordance at similar levels of divergence (e.g., *N. arge* 1 with subclades in West‐Central vs. East‐Central Africa, and *N. tragata* + *N. javanica*). This raises the possibility that the pattern results from sex‐biased dispersal within the *N. thebaica* species group. Monadjem (2005) longitudinal study of *N. thebaica* survivorship in Swaziland offers robust evidence for female philopatry and male‐biased dispersal. Of 39 females he banded as adults, nearly a quarter were living in the same culverts 4.5 years later, whereas only one of the 29 banded males was recaptured. Although other *Nycteris* dispersal studies are lacking, his observations are compatible with the strongly contrasting mitochondrial and nuclear population structures inferred here and warrant further life‐history studies of other *Nycteris* species. However, analyses using microsatellites or SNPs to exclude other possible explanations for this mitonuclear discordance would be necessary to establish this.

### 4.2 Phylogenetic relationships

Our analyses conflict with earlier efforts to resolve the phylogenetic relationships of *Nycteris*. The tree of Shi and Rabosky (2015) recovered the pair *N. hispida* and *N. thebaica* as sister to all *Nycteris* species; the remainder were arranged as *N. javanica* + *N. tragata* as sister to *N. grandis + N. arge,* with *N. macrotis* subtending this group. In contrast, Amador et al. (2018) recovered *N. macrotis* as the earliest diverging lineage of *Nycteris,* which was sister to a pair of clades, one containing the Asian species *N. tragata* and *N. javanica* and the other containing the African species *N. grandis* and *N. arge* as sisters, joined successively by *N. hispida* and *N. thebaica*. The two studies used the same 7 *Nycteris* species (*arge*, *grandis*, *hispida*, *javanica*, *macrotis*, *thebaica*, and *tragata*), but Amador et al. (2018) partitioned *cytb* and the two nuclear genes included in their analysis (vWF and BRCA) by codon position, whereas Shi and Rabosky partitioned their dataset by gene. All 7 *Nycteris* species in the concatenated ML analysis of Shi and Rabosky had BS support ≥70%, whereas the concatenated ML tree of Amador et al. (2018) more weakly supported *N. macrotis* as sister to the remaining *Nycteris* clades at 60%.

In contrast to both studies, we found strong support (PP 1.0) for two major subclades within the genus (Figures 4 and 5), each comprised of two groups of species. In the first subclade, *N. thebaica* and the three *N.* cf. *thebaica* clades form one group (Clade 1A), while *N. arge* clade 2 and the two *N. nana* clades comprise their sister (Clade 1B). In the second subclade, three *N. macrotis* clades comprise one group (Clade 2B) and *N. tragata*, *N.* *grandis*, *N. hispida/aurita,* and *N.* cf. *hispida/aurita* comprise the other (Clade 2A). Less securely placed in the latter group is *N. arge* 1 (PP = 0.84). Additional highly informative nuclear markers for bats (e.g., Dool et al., 2016; Demos et al., 2018) are likely responsible for improved resolution although better taxonomic and geographic sampling in this study may also contribute. To some extent, comparisons with these earlier investigations are limited by our conservative approach in withholding species assignment for specimens deemed cryptic and/or subtly differentiated from named taxa. That said, expanded taxonomic coverage alone, regardless of names assigned to terminals in the study, could be expected to result in conflicting topologies, as would possible incorrect species identifications from previous studies that relied on GenBank data.

Comparing the mitochondrial (Figure 2a), concatenated nuclear (Figure 4), and species trees (Figure 5) in our analyses, the only major inconsistency concerns the position of *N. arge* 2 + *N. nana* 1 + *N. nana* 2. The *cytb* gene tree analyses strongly support this clade as sister to *N. macrotis*, but the high genetic distances in this dataset raise the specter of substitutional saturation. In turn, the concatenated gene tree analyses infer poor support for the clade as sister to *N. thebaica*, whereas the species tree analyses strongly support the clade as sister to the *N. thebaica* group (PP = 1.0). Examination of relationships in both the concatenated nuclear and species trees, along with their substantial branch lengths, provide strong support for two major and four subordinate clades of species within *Nycteris*. The subordinate groupings represent species groups, as discussed below. The major clades have not previously been recognized, and the use of subgenera for these clades may be appropriate. As discussed by Teta (2019), there are several advantages of applying the category of subgenus to well‐supported clades. The category is recognized in zoological nomenclature at a rank intermediate between genus and species and regulated by the zoological code. Its use preserves binomial usage, and thus nomenclatural stability, and by joining closely related species it can be used to generate phylogenetic predictions (e.g., Teta, Cañón, Patterson, & Pardiñas, 2017; Voss, Gutiérrez, Solari, Rossi, & Jansa, 2014). Proposals to formally name these groups of *Nycteris* species should include the compilation of comprehensive morphological diagnoses, which is outside the purview of this study.

### 4.3 Species groups of *Nycteris*

The four subordinate clusters in the two subclades have been recognized since Andersen's (1912) first generic synopsis. Except for the position of the Asian taxa, they roughly correspond to his four species groups as they are currently defined (e.g., Happold, 2013b). All are separated by *cytb* distances of at least 16%, and their clade membership is strongly supported in the species tree. First, the cluster comprising *Nycteris thebaica* + *N.* cf. *thebaica* 1–3 (Clade 1A) is strongly supported as monophyletic in the species tree and is >17% *cytb* diverged from its sister. This group is distributed in northeastern, eastern, and southern Africa and, by definition, corresponds to the *N. thebaica* species group, although other assigned group members *N. gambiensis* and *N. vinsoni* were not explicitly included in our analyses. Second, and sister to the *N. thebaica* species group, is a cluster comprising *N. arge* 2 + *N. nana* 1 and 2 (Clade 1B), which is strongly supported as monophyletic and genetically distant (>17% *cytb*) from all other *Nycteris*. Distributed across western, Central, and eastern Africa, this grouping corresponds to the *arge* species group, although our analyses failed to include other group members *N. intermedia* and *N. major* (unless the former is in fact represented but mislabeled as *N. nana* 1 or *N. nana* 2). Third, the cluster comprising *N. hispida/aurita*, *N.* cf. *hispida/aurita*, *N. grandis*, and *N. tragata* (Clade 2A) is strongly supported as monophyletic and is >16% *cytb* diverged from the *N. macrotis* lineages that comprise its sister. This group is widely distributed; its African members correspond to the *N. hispida* species group but there is strong support for the additional membership of *N. tragata* from SE Asia. Although we did not sequence *N. javanica* for nuclear loci, the close relationship of *N. javanica* to *N. tragata* is well established (Amador et al., 2018; Shi & Rabosky, 2015; Figure 2a). Previous morphological indications that *N. javanica* and *N. tragata* were sister to the *N. thebaica, N. hispida*, and *N. macrotis* species groups (Griffiths, 1997) were clearly homoplasious. The relationship of *N. arge* 1 is uncertain, although it is weakly supported as sister to clade 2A in the species tree. Fourth, a final cluster comprises *N. macrotis* clades 1–3 (Clade 2B) and is strongly supported as monophyletic. It is >16% *cytb* diverged from its sister clade and includes members from South Sudan to Malawi and Mozambique east of the Albertine Rift and Congo Basin. It corresponds to the *macrotis* group, although our samples did not include identified representatives of *N. madagascariensis*, *N. parisii*, and *N. woodi*.

The fact that every newly sequenced *Nycteris* is associated with an identifiable museum voucher specimen means that forging linkages between genetic and morphological patterns is possible and because *Nycteris* taxa were all proposed on morphological grounds, this linkage enables sound nomenclature. Had the same genetic work been accomplished with biopsies from bats that were subsequently released, which is now technically possible, it would be impossible to confirm the identities and characterize the distinctive features of these lineages. As a case in point, lineages designated *N. arge *clades 1 and 2 (Figures 4 and 5) were each identified as *N. arge* in the field but clearly represent distinct lineages that likely belong to different species groups. Resolving the relationships of cryptic lineages is greatly expedited by comprehensive voucher material that preserves a broad array of biological characters, in the case of bats including skeletal and soft‐part anatomy, genitalia, vocalizations, and parasites, in addition to their genetic attributes (Gippoliti, 2018). Currently, 16 species of *Nycteris* are accepted as valid species, but several of these lack tissue samples in repositories or GenBank accessions and many lack vouchers with genetic material from near their type localities, hindering efforts to specify names (see Figure S1 in Supporting Information). Based on the number of well‐supported and deeply diverged lineages inferred here using multiple datasets and phylogenetic inference methods, it is likely that our analyses have uncovered several undescribed taxa.

The next steps in elucidating Nycteridae relationships will be in reconciling the phylogenetic patterns described in this paper with the extensive morphological analyses developed around *Nycteris* types and throughout their geographic distributions by Van Cakenberghe and de Vree (1985, 1993a, 1993b, 1998). Only then will it be possible to replace the various annotations on our figures with a robust binomial nomenclature.

## Supporting information


**Table S1.** List of locality data for specimens used in genetic analyses of *Nycteris*.
**Figure S1.** Geographic sampling of genetic data used in this study. Plotting symbols denote the locations of one or more individuals represented by mitochondrial sequence (*cytb*) downloaded from GenBank (+), those represented only by *cytb* data newly generated for this study (open circles), and those where both mitochondrial and nuclear sequences were newly generated (filled circles). Taxon, localities, and coordinates for these points are included in Supporting Information Table S1.
**Figure S2.** Species tree inferred in StarBEAST for *Nycteris* for 21 clades, including *Nycteris thebaica* clades 1 to 6. Nodes are labeled with posterior probabilities.

## APPENDIX 1

List of specimens used in genetic analyses of *Nycteris*. Taxon names, voucher numbers, and GenBank accession numbers of sampled individuals of *Nycteris*: FMNH — Field Museum of Natural History, Chicago; LSUMZ — Louisiana State University, Museum of Natural Science; MHNG — Muséum d'Histoire Naturelle, Genève; NMK — National Museums of Kenya, Nairobi; ROM — Royal Ontario Museum, Toronto; TTU — Museum of Texas Tech University, Lubbock.

**Table:**

Taxon	Voucher No.	*cytb*	*ACOX2*	*COPS7A*	*ROGDI*	*STAT5A*
*Coleura afra*	FMNH 220403	MK837103	MK837325	MK837394	MK837464	MK837534
*Nycteris arge* 1	FMNH 167763	MK837079	MK837329	MK837398	MK837468	MK837538
*Nycteris arge* 1	FMNH 222429	MK837077	MK837327	MK837396	MK837466	MK837536
*Nycteris arge* 1	FMNH 226934	MK837078	MK837328	MK837397	MK837467	MK837537
*Nycteris arge* 1	FMNH 227433	MK837076	MK837326	MK837395	MK837465	MK837535
*Nycteris arge* 1	FMNH 232918	MK837080				
*Nycteris arge* 2	FMNH 149405	MK837081	MK837330	MK837399	MK837469	MK837539
*Nycteris arge* 2	FMNH 215539	MK837083				
*Nycteris arge* 2	FMNH 215540	MK837084				
*Nycteris arge* 2	FMNH 222430	MK837082				
*Nycteris arge* 2	FMNH 224102	MK837088	MK837332	MK837401	MK837471	MK837541
*Nycteris arge* 2	FMNH 224103	MK837089				
*Nycteris arge* 2	FMNH 224104	MK837090				
*Nycteris arge* 2	NMK 184961	MK837085	MK837331	MK837400	MK837470	MK837540
*Nycteris arge* 2	NMK 184967	MK837086				
*Nycteris arge* 2	NMK 187405	MK837087				
*Nycteris* cf. *hispida/aurita*	FMNH 187139	MK837094	MK837335	MK837404	MK837474	MK837544
*Nycteris* cf. *hispida/aurita*	FMNH 220978	MK837091	MK837333	MK837402	MK837472	MK837542
*Nycteris* cf. *hispida/aurita*	FMNH 220979	MK837092	MK837334	MK837403	MK837473	MK837543
*Nycteris* cf. *hispida/aurita*	FMNH 220982	MK837093				
*Nycteris* cf. *thebaica* 1	FMNH 195603	MK837096	MK837337	MK837406	MK837476	MK837546
*Nycteris* cf. *thebaica* 1	FMNH 195604	MK837097				
*Nycteris* cf. *thebaica* 1	FMNH 195605	MK837098	MK837338	MK837407	MK837477	MK837547
*Nycteris* cf. *thebaica* 1	FMNH 195606	MK837099				
*Nycteris* cf. *thebaica* 1	FMNH 226239	MK837095	MK837336	MK837405	MK837475	MK837545
*Nycteris* cf. *thebaica* 2	NMK 184231	MK837100	MK837339	MK837408	MK837478	MK837548
*Nycteris* cf. *thebaica* 2	NMK 184384	MK837101	MK837340	MK837409	MK837479	MK837549
*Nycteris* cf. *thebaica* 3	FMNH 158336	MK837102	MK837341	MK837410	MK837480	MK837550
*Nycteris grandis*	FMNH 150065	MK837111				
*Nycteris grandis*	FMNH 151187	MK837112	MK837346	MK837415	MK837485	MK837555
*Nycteris grandis*	FMNH 151188	MK837113				
*Nycteris grandis*	FMNH 151189	MK837114				
*Nycteris grandis*	FMNH 151190	MK837115				
*Nycteris grandis*	FMNH 151416	MK837116				
*Nycteris grandis*	FMNH 168092	MK837117				
*Nycteris grandis*	FMNH 192814	MK837118				
*Nycteris grandis*	FMNH 192815	MK837119				
*Nycteris grandis*	FMNH 192816	MK837120				
*Nycteris grandis*	FMNH 192882	MK837121				
*Nycteris grandis*	FMNH 192883	MK837122	MK837347	MK837416	MK837486	MK837556
*Nycteris grandis*	FMNH 192884	MK837123				
*Nycteris grandis*	FMNH 192885	MK837124				
*Nycteris grandis*	FMNH 192936	MK837125				
*Nycteris grandis*	FMNH 213625	MK837110	MK837345	MK837414	MK837484	MK837554
*Nycteris grandis*	FMNH 219603	MK837107	MK837343	MK837412	MK837482	MK837552
*Nycteris grandis*	FMNH 222427	MK837108	MK837344	MK837413	MK837483	MK837553
*Nycteris grandis*	FMNH 222428	MK837109				
*Nycteris grandis*	FMNH 227439	MK837104				
*Nycteris grandis*	FMNH 227441	MK837105	MK837342	MK837411	MK837481	MK837551
*Nycteris grandis*	FMNH 227442	MK837106				
*Nycteris hispida/aurita*	FMNH 137625	MK837140				
*Nycteris hispida/aurita*	FMNH 137626	MK837141				
*Nycteris hispida/aurita*	FMNH 151191	MK837139	MK837352	MK837421	MK837491	MK837561
*Nycteris hispida/aurita*	FMNH 165131	MK837142				
*Nycteris hispida/aurita*	FMNH 195607	MK837138	MK837351	MK837420	MK837490	MK837560
*Nycteris hispida/aurita*	FMNH 215546	MK837130				
*Nycteris hispida/aurita*	FMNH 215547	MK837131				
*Nycteris hispida/aurita*	FMNH 215548	MK837132				
*Nycteris hispida/aurita*	FMNH 220746	MK837133				
*Nycteris hispida/aurita*	FMNH 220980	MK837127				
*Nycteris hispida/aurita*	FMNH 225217	MK837135				
*Nycteris hispida/aurita*	FMNH 225218	MK837136				
*Nycteris hispida/aurita*	FMNH 225240	MK837137	MK837350	MK837419	MK837489	MK837559
*Nycteris hispida/aurita*	FMNH 225445	MK837134				
*Nycteris hispida/aurita*	FMNH 227445	MK837126	MK837348	MK837417	MK837487	MK837557
*Nycteris hispida/aurita*	FMNH 232892	MK837143				
*Nycteris hispida/aurita*	FMNH 232893	MK837144				
*Nycteris hispida/aurita*	FMNH 232902	MK837145				
*Nycteris hispida/aurita*	FMNH 232903	MK837146	MK837353	MK837422	MK837492	MK837562
*Nycteris hispida/aurita*	FMNH 232904	MK837317				
*Nycteris hispida/aurita*	FMNH 232905	MK837147				
*Nycteris hispida/aurita*	FMNH 232906	MK837148				
*Nycteris hispida/aurita*	FMNH 232908	MK837149				
*Nycteris hispida/aurita*	MHNG 1971.039	HQ693722				
*Nycteris hispida/aurita*	MHNG 1971.04	HQ693723				
*Nycteris hispida/aurita*	NMK 184937	MK837128				
*Nycteris hispida/aurita*	NMK 184976	MK837129	MK837349	MK837418	MK837488	MK837558
*Nycteris javanica*	ROM 101970	EF584225				
*Nycteris macrotis* 1	FMNH 192937	MK837170				
*Nycteris macrotis* 1	FMNH 215541	MK837150	MK837354	MK837423	MK837493	MK837563
*Nycteris macrotis* 1	FMNH 216029	MK837151				
*Nycteris macrotis* 1	FMNH 216030	MK837152	MK837355	MK837424	MK837494	MK837564
*Nycteris macrotis* 1	FMNH 216031	MK837153				
*Nycteris macrotis* 1	FMNH 216032	MK837154				
*Nycteris macrotis* 1	FMNH 216033	MK837155				
*Nycteris macrotis* 1	FMNH 216034	MK837160				
*Nycteris macrotis* 1	FMNH 216035	MK837161	MK837356	MK837425	MK837495	MK837565
*Nycteris macrotis* 1	FMNH 216036	MK837158				
*Nycteris macrotis* 1	FMNH 219068	MK837171				
*Nycteris macrotis* 1	FMNH 219069	MK837172	MK837359	MK837428	MK837498	MK837568
*Nycteris macrotis* 1	FMNH 219239	MK837173				
*Nycteris macrotis* 1	FMNH 220492	MK837156				
*Nycteris macrotis* 1	FMNH 220494	MK837157				
*Nycteris macrotis* 1	FMNH 220742	MK837318				
*Nycteris macrotis* 1	FMNH 220744	MK837162				
*Nycteris macrotis* 1	FMNH 220745	MK837319				
*Nycteris macrotis* 1	FMNH 220977	MK837163	MK837357	MK837426	MK837496	MK837566
*Nycteris macrotis* 1	FMNH 220981	MK837159				
*Nycteris macrotis* 1	FMNH 223200	MK837174				
*Nycteris macrotis* 1	FMNH 223660	MK837175				
*Nycteris macrotis* 1	FMNH 232911	MK837176				
*Nycteris macrotis* 1	FMNH 232912	MK837177	MK837360	MK837429	MK837499	MK837569
*Nycteris macrotis* 1	FMNH 232913	MK837178				
*Nycteris macrotis* 1	FMNH 232914	MK837179				
*Nycteris macrotis* 1	FMNH 232915	MK837180				
*Nycteris macrotis* 1	FMNH 232916	MK837320				
*Nycteris macrotis* 1	FMNH 232917	MK837181	MK837361	MK837430	MK837500	MK837570
*Nycteris macrotis* 1	FMNH HB115	MK837166	MK837358	MK837427	MK837497	MK837567
*Nycteris macrotis* 1	FMNH HB121	MK837167				
*Nycteris macrotis* 1	FMNH HB122	MK837168				
*Nycteris macrotis* 1	FMNH HB124	MK837169				
*Nycteris macrotis* 1	FMNH HB61	MK837164				
*Nycteris macrotis* 1	FMNH HB62	MK837165				
*Nycteris macrotis* 2	FMNH 220739	MK837182				
*Nycteris macrotis* 2	FMNH 220740	MK837321	MK837362	MK837431	MK837501	MK837571
*Nycteris macrotis* 3	FMNH 226237	MK837183	MK837363	MK837432	MK837502	MK837572
*Nycteris macrotis* 3	FMNH 228897	MK837184	MK837364	MK837433	MK837503	MK837573
*Nycteris macrotis* 3	FMNH 228898	MK837185	MK837365	MK837434	MK837504	MK837574
*Nycteris macrotis* 3	FMNH 228899	MK837186				
*Nycteris macrotis* 3	FMNH 228900	MK837187				
*Nycteris macrotis* 3	FMNH 228901	MK837188				
*Nycteris nana* 1	FMNH 227448	MK837189	MK837366	MK837435	MK837505	MK837575
*Nycteris nana* 2	FMNH 167764	MK837191	MK837368	MK837437	MK837507	MK837577
*Nycteris nana* 2	FMNH 227446	MK837190	MK837367	MK837436	MK837506	MK837576
*Nycteris thebaica* 1	FMNH 225210	MK837236				
*Nycteris thebaica* 1	FMNH 225241	MK837237	MK837372	MK837441	MK837511	MK837581
*Nycteris thebaica* 1	FMNH 225242	MK837238	MK837373	MK837442	MK837512	MK837582
*Nycteris thebaica* 1	FMNH 225243	MK837239				
*Nycteris thebaica* 1	FMNH 225244	MK837240				
*Nycteris thebaica* 1	FMNH 225245	MK837241	MK837374	MK837443	MK837513	MK837583
*Nycteris thebaica* 1	FMNH 225406	MK837192				
*Nycteris thebaica* 1	FMNH 225407	MK837193				
*Nycteris thebaica* 1	FMNH 225408	MK837194				
*Nycteris thebaica* 1	FMNH 225409	MK837195				
*Nycteris thebaica* 1	FMNH 225410	MK837196				
*Nycteris thebaica* 1	FMNH 225411	MK837197				
*Nycteris thebaica* 1	FMNH 225412	MK837198				
*Nycteris thebaica* 1	FMNH 225413	MK837199				
*Nycteris thebaica* 1	FMNH 225420	MK837200				
*Nycteris thebaica* 1	FMNH 225421	MK837201				
*Nycteris thebaica* 1	FMNH 225423	MK837202				
*Nycteris thebaica* 1	FMNH 225424	MK837203				
*Nycteris thebaica* 1	FMNH 225425	MK837204				
*Nycteris thebaica* 1	FMNH 225426	MK837205				
*Nycteris thebaica* 1	FMNH 225442	MK837206	MK837369	MK837438	MK837508	MK837578
*Nycteris thebaica* 1	FMNH 225443	MK837207				
*Nycteris thebaica* 1	FMNH 225444	MK837208				
*Nycteris thebaica* 1	FMNH 225446	MK837209				
*Nycteris thebaica* 1	FMNH 225447	MK837210				
*Nycteris thebaica* 1	FMNH 225448	MK837211				
*Nycteris thebaica* 1	FMNH 225449	MK837212				
*Nycteris thebaica* 1	FMNH 225450	MK837213				
*Nycteris thebaica* 1	FMNH 225451	MK837214				
*Nycteris thebaica* 1	FMNH 225452	MK837215				
*Nycteris thebaica* 1	FMNH 225453	MK837216				
*Nycteris thebaica* 1	FMNH 225454	MK837217				
*Nycteris thebaica* 1	FMNH 225455	MK837218				
*Nycteris thebaica* 1	FMNH 225456	MK837219				
*Nycteris thebaica* 1	FMNH 225457	MK837220				
*Nycteris thebaica* 1	FMNH 225458	MK837221	MK837370	MK837439	MK837509	MK837579
*Nycteris thebaica* 1	FMNH 225459	MK837222				
*Nycteris thebaica* 1	FMNH 225460	MK837223				
*Nycteris thebaica* 1	FMNH 225461	MK837224				
*Nycteris thebaica* 1	FMNH 225462	MK837225				
*Nycteris thebaica* 1	FMNH 225463	MK837226				
*Nycteris thebaica* 1	FMNH 225464	MK837227				
*Nycteris thebaica* 1	FMNH 225465	MK837322				
*Nycteris thebaica* 1	FMNH 225466	MK837228				
*Nycteris thebaica* 1	FMNH 225467	MK837229				
*Nycteris thebaica* 1	FMNH 225468	MK837230				
*Nycteris thebaica* 1	FMNH 225469	MK837323				
*Nycteris thebaica* 1	FMNH 225470	MK837231				
*Nycteris thebaica* 1	FMNH 225471	MK837232				
*Nycteris thebaica* 1	FMNH 225472	MK837233	MK837371	MK837440	MK837510	MK837580
*Nycteris thebaica* 1	FMNH 225473	MK837234				
*Nycteris thebaica* 1	FMNH 225474	MK837235				
*Nycteris thebaica* 2	FMNH 147220	MK837242	MK837375	MK837444	MK837514	MK837584
*Nycteris thebaica* 2	FMNH 198085	MK837243	MK837376	MK837445	MK837515	MK837585
*Nycteris thebaica* 2	FMNH 198086	MK837244				
*Nycteris thebaica* 2	FMNH 198087	MK837245				
*Nycteris thebaica* 2	FMNH 198088	MK837246	MK837377	MK837446	MK837516	MK837586
*Nycteris thebaica* 2	FMNH 198089	MK837247				
*Nycteris thebaica* 3	FMNH 215536	MK837324				
*Nycteris thebaica* 3	FMNH 215537	MK837249				
*Nycteris thebaica* 3	FMNH 215538	MK837250	MK837378	MK837447	MK837517	MK837587
*Nycteris thebaica* 3	FMNH 215542	MK837259				
*Nycteris thebaica* 3	FMNH 215543	MK837260				
*Nycteris thebaica* 3	FMNH 215544	MK837261				
*Nycteris thebaica* 3	FMNH 215545	MK837262				
*Nycteris thebaica* 3	FMNH 220741	MK837258				
*Nycteris thebaica* 3	FMNH 225400	MK837263				
*Nycteris thebaica* 3	FMNH 225401	MK837264				
*Nycteris thebaica* 3	FMNH 225402	MK837265				
*Nycteris thebaica* 3	FMNH 225403	MK837266	MK837381	MK837450	MK837520	MK837590
*Nycteris thebaica* 3	FMNH 225404	MK837267				
*Nycteris thebaica* 3	FMNH 225405	MK837268				
*Nycteris thebaica* 3	FMNH 232109	MK837277				
*Nycteris thebaica* 3	FMNH 232429	MK837278	MK837383	MK837452	MK837522	MK837592
*Nycteris thebaica* 3	FMNH 232430	MK837279				
*Nycteris thebaica* 3	FMNH 232431	MK837280				
*Nycteris thebaica* 3	FMNH 232919	MK837281				
*Nycteris thebaica* 3	NMK 184407	MK837253	MK837379	MK837448	MK837518	MK837588
*Nycteris thebaica* 3	NMK 184520	MK837254	MK837380	MK837449	MK837519	MK837589
*Nycteris thebaica* 3	NMK 184521	MK837255				
*Nycteris thebaica* 3	NMK 184522	MK837256				
*Nycteris thebaica* 3	NMK 184636	MK837257				
*Nycteris thebaica* 3	NMK 184658	MK837248				
*Nycteris thebaica* 3	NMK 184759	MK837269				
*Nycteris thebaica* 3	NMK 184854	MK837270				
*Nycteris thebaica* 3	NMK 184855	MK837271				
*Nycteris thebaica* 3	NMK 185133	MK837252				
*Nycteris thebaica* 3	NMK 187337	MK837272	MK837382	MK837451	MK837521	MK837591
*Nycteris thebaica* 3	NMK 187338	MK837273				
*Nycteris thebaica* 3	NMK 187339	MK837274				
*Nycteris thebaica* 3	NMK 187340	MK837275				
*Nycteris thebaica* 3	NMK 187341	MK837276				
*Nycteris thebaica* 3	NMK 187450	MK837251				
*Nycteris thebaica* 4	FMNH 216037	MK837282	MK837386	MK837455	MK837525	MK837595
*Nycteris thebaica* 4	FMNH 216039	MK837283		MK837456	MK837526	MK837596
*Nycteris thebaica* 4	FMNH 216040	MK837284				
*Nycteris thebaica* 4	FMNH 216042	MK837285				
*Nycteris thebaica* 4	FMNH 216043	MK837286				
*Nycteris thebaica* 4	FMNH 220446	MK837288				
*Nycteris thebaica* 4	FMNH 220447	MK837287				
*Nycteris thebaica* 4	FMNH 220448	MK837289				
*Nycteris thebaica* 4	FMNH 220449	MK837290				
*Nycteris thebaica* 4	FMNH 220450	MK837291				
*Nycteris thebaica* 4	FMNH 220451	MK837292				
*Nycteris thebaica* 4	FMNH 220452	MK837293				
*Nycteris thebaica* 4	FMNH 220453	MK837294	MK837384	MK837453	MK837523	MK837593
*Nycteris thebaica* 4	FMNH 220455	MK837295				
*Nycteris thebaica* 4	FMNH 220484	MK837296	MK837385	MK837454	MK837524	MK837594
*Nycteris thebaica* 4	FMNH 220485	MK837297				
*Nycteris thebaica* 4	FMNH 220486	MK837298				
*Nycteris thebaica* 4	FMNH 220487	MK837299				
*Nycteris thebaica* 4	FMNH 220488	MK837300				
*Nycteris thebaica* 5	FMNH 213626	MK837301	MK837387	MK837457	MK837527	MK837597
*Nycteris thebaica* 5	FMNH 213627	MK837302	MK837388	MK837458	MK837528	MK837598
*Nycteris thebaica* 5	FMNH 213628	MK837303	MK837389	MK837459	MK837529	MK837599
*Nycteris thebaica* 5	FMNH 213629	MK837304				
*Nycteris thebaica* 5	FMNH 213630	MK837305				
*Nycteris thebaica* 5	FMNH 213631	MK837306				
*Nycteris thebaica* 5	FMNH 213632	MK837307				
*Nycteris thebaica* 6	FMNH 187360	MK837310				
*Nycteris thebaica* 6	FMNH 187361	MK837311	MK837391	MK837461	MK837531	MK837601
*Nycteris thebaica* 6	FMNH 187412	MK837312				
*Nycteris thebaica* 6	FMNH 193210	MK837313				
*Nycteris thebaica* 6	FMNH 219066	MK837314				
*Nycteris thebaica* 6	FMNH 219067	MK837315	MK837392	MK837462	MK837532	MK837602
*Nycteris thebaica* 6	FMNH 226238	MK837308	MK837390	MK837460	MK837530	MK837600
*Nycteris thebaica* 6	FMNH 226240	MK837309				
*Nycteris tragata*	LSUMZ 4413	MK837316	MK837393	MK837463	MK837533	MK837603
*Nycteris tragata*	TTU 108180	EU21624				
