# Supplementary material for: Molecular phylogenetics of slit‐faced bats (Chiroptera: Nycteridae) reveal deeply divergent African lineages
Source: J Zool Syst Evol Res. 2019 Aug 20;57(4):1019–38. doi: 10.1111/jzs.12313 (PMC6919933; doi:10.1111/jzs.12313)

Demos T.C., Webala P.W., Kerbis Peterhans J.C., Goodman S.M., Bartonjo M., and Patterson B.D. Molecular phylogenetics of slit-faced bats (Chiroptera: Nycteridae) reveals deeply divergent African lineages. *Journal of Zoological Systematics and Evolutionary Research*.

Supplemental Information Table S1. List of specimens used in genetic analyses of *Nycteris*. Specimen details, localities, and GenBank accession numbers of sampled individuals of *Nycteris*: FMNH — Field Museum of Natural History, Chicago; LSUMZ — Louisiana State University, Museum of Natural Science; MHNG — Muséum d'Histoire Naturelle, Genève; NMK — National Museums of Kenya, Nairobi; ROM — Royal Ontario Museum, Toronto; TTU — Museum of Texas Tech University, Lubbock.

**Taxon**	**Voucher No.**	**Country**	**County**	**Locality**	**Latitude**	**Longitude**
Nycteris arge 1	FMNH 227433	Central African Republic		Parc National de Dzanga-Ndoki, Mabea Bai, 21.4 kms NE Bayanga	3.0333	16.41
Nycteris arge 1	FMNH 167763	Gabon		Réserve de la Moukalaba, 12.2 km NW Doussala	-2.283333	10.49722
Nycteris arge 1	FMNH 232918	Uganda		Budongo CFR, Budongo Siba block	1.706146	31.48824
Nycteris arge 1	FMNH 222429	Democratic Republic of Congo		14 km by road N Boende, Quatorze	0.16764	20.92603
Nycteris arge 1	FMNH 226934	Democratic Republic of Congo		Katopa, west side of Lomani River (left bank)	-2.747295	25.10126
Nycteris arge 2	NMK 184961	Kenya	Kakamega	Kakamega Forest National Reserve, Buzambuli Trail 1	0.35006	34.86141
Nycteris arge 2	NMK 184967	Kenya	Kakamega	Kakamega Forest, Ikhondo Junction	0.35293	34.86201
Nycteris arge 2	NMK 187405	Kenya	Kakamega	Kakamega Forest, Colobus Circuit 1	0.3561	34.86135
Nycteris arge 2	FMNH 215539	Kenya	Kakamega	Kakamega National Reserve, Buyangu Village	0.3523	34.86469
Nycteris arge 2	FMNH 215540	Kenya	Kakamega	Kakamega National Reserve, Buyangu Village	0.3523	34.86469
Nycteris arge 2	FMNH 224102	Uganda		Kibale Forest NP, Ngogo, 24 km SE of Fort Portal	30.0628	30.42608
Nycteris arge 2	FMNH 224103	Uganda		Kibale Forest NP, Ngogo, 24 km SE of Fort Portal	30.0628	30.42608
Nycteris arge 2	FMNH 224104	Uganda		Kibale Forest NP, Ngogo, 24 km SE of Fort Portal	30.0628	30.42608
Nycteris arge 2	FMNH 222430	Democratic Republic of Congo		14 km by road N Boende, Quatorze	0.16764	20.92603
Nycteris arge 2	FMNH 149405	Democratic Republic of Congo		Epulu, Epulu R, right bank	-1.4166	28.5833
Nycteris cf. *hispida/aurita*	FMNH 220978	Kenya	Kajiado	Amboseli National Park, Amboseli Serena Lodge	-2.70548	37.26607
Nycteris cf. *hispida/aurita*	FMNH 220979	Kenya	Kajiado	Amboseli National Park, Amboseli Serena Lodge	-2.70548	37.26607
Nycteris cf. *hispida/aurita*	FMNH 220982	Kenya	Kajiado	Amboseli National Park, Amboseli Serena Lodge	-2.70548	37.26607
Nycteris cf. *hispida/aurita*	FMNH 187139	Tanzania		Tarangire National Park, Foley's Camp	-3.79785	36.06882
Nycteris cf. *thebaica* 1	FMNH 226239	Malawi		Mangochi Hills, Skull Rock Scout Camp	-14.50872	35.23344
Nycteris cf. *thebaica* 1	FMNH 195603	South Africa		Marloth Park, Lionspruit	-25.35819	31.79019

Nycteris cf. *thebaica* 1	FMNH 195604	South Africa		Marloth Park, Lionspruit	-25.35819	31.79019
Nycteris cf. *thebaica* 1	FMNH 195605	South Africa		Marloth Park, Lionspruit	-25.35819	31.79019
Nycteris cf. *thebaica* 1	FMNH 195606	South Africa		Marloth Park, Lionspruit	-25.35819	31.79019
Nycteris cf. *thebaica* 2	NMK 184384	Kenya	Marsabit	Marsabit National Park and Reserve, 6.07 km SW campground near headquarters (NETSI)	2.28311	37.95428
Nycteris cf. *thebaica* 2	NMK 184231	Kenya	Marsabit	Marsabit National Park and Reserve, campground near headquarters, officers housing	2.32036	37.99402
Nycteris cf. *thebaica* 3	FMNH 158336	Tanzania		Uluguru Mts, Uluguru North Forest Reserve, 3 km W, 1.3 km N Tegetero	-6.929167	37.70556
Nycteris grandis	FMNH 227439	Central African Republic		Parc National de Dzanga-Ndoki, Mabea Bai, 21.4 kms NE Bayanga	3.0333	16.41
Nycteris grandis	FMNH 227441	Central African Republic		Parc National de Dzanga-Ndoki, Mabea Bai, 21.4 kms NE Bayanga	3.0333	16.41
Nycteris grandis	FMNH 227442	Central African Republic		Parc National de Dzanga-Ndoki, Mabea Bai, 21.4 kms NE Bayanga	3.0333	16.41
Nycteris grandis	FMNH 213625	Mozambique		near Mossuril, near ruins of Portugese ruler house	-14.96778	40.66389
Nycteris grandis	FMNH 150065	Tanzania		East Usambara Mts, 4.5 km WNW Amani, Monga Tea Estate	-5.1	38.6
Nycteris grandis	FMNH 151416	Tanzania		East Usambara Mts, 4.5 km WNW Amani, Monga Tea Estate	-5.1	38.6
Nycteris grandis	FMNH 168092	Tanzania		East Usambara Mts, 4.5 km WNW Amani, Monga Tea Estate	-5.1	38.6
Nycteris grandis	FMNH 151187	Tanzania		Gonja Forest Reserve, 2 km SW Gonja Maore	-4.2667	38.0333
Nycteris grandis	FMNH 151188	Tanzania		Gonja Forest Reserve, 2 km SW Gonja Maore	-4.2667	38.0333
Nycteris grandis	FMNH 151189	Tanzania		Gonja Forest Reserve, 2 km SW Gonja Maore	-4.2667	38.0333
Nycteris grandis	FMNH 151190	Tanzania		Gonja Forest Reserve, 2 km SW Gonja Maore	-4.2667	38.0333
Nycteris grandis	FMNH 192936	Tanzania		Minziro Forest	-1.094181	31.51538
Nycteris grandis	FMNH 192814	Tanzania		Ngezi Forest	-4.942739	39.70823
Nycteris grandis	FMNH 192815	Tanzania		Ngezi Forest	-4.942739	39.70823
Nycteris grandis	FMNH 192816	Tanzania		Ngezi Forest	-4.941761	39.71035
Nycteris grandis	FMNH 192883	Tanzania		Ngezi Forest, Chonja village	-4.950581	39.72918
Nycteris grandis	FMNH 192884	Tanzania		Ngezi Forest, Chonja village	-4.950581	39.72918

Nycteris grandis	FMNH 192885	Tanzania		Ngezi Forest, Chonja village	-4.950581	39.72918
Nycteris grandis	FMNH 192882	Tanzania		Ngezi Forest, Kibatini village	-4.956569	39.71779
Nycteris grandis	FMNH 222427	Democratic Republic of Congo		14 km by road N Boende, Quatorze	0.16764	20.92603
Nycteris grandis	FMNH 222428	Democratic Republic of Congo		14 km by road N Boende, Quatorze	0.16764	20.92603
Nycteris grandis	FMNH 219603	Democratic Republic of Congo		Baliko	0.24128	20.8833
Nycteris hispida/aurita	FMNH 227445	Central African Republic		Parc National de Dzanga-Ndoki, Mabea Bai, 21.4 kms NE Bayanga	3.0333	16.41
Nycteris hispida/aurita	FMNH 220980	Kenya	Kajiado	Amboseli Elephant Research Camp	-2.6789	37.26656
Nycteris hispida/aurita	FMNH 215546	Kenya	Kisimu	Kisimu Impala Sanctuary, State Lodge Campsite	-0.10961	34.74593
Nycteris hispida/aurita	FMNH 215547	Kenya	Kisimu	Kisimu Impala Sanctuary, State Lodge Campsite	-0.10961	34.74593
Nycteris hispida/aurita	FMNH 215548	Kenya	Kisimu	Kisimu Impala Sanctuary, State Lodge Campsite	-0.10961	34.74593
Nycteris hispida/aurita	FMNH 225445	Kenya	Narok	Masai Mara National Reserve, Sarova Mara Lodge	-1.53105	35.32022
Nycteris hispida/aurita	FMNH 220746	Kenya	Meru	Meru National Park, Murera Gate guardhouse	0.267933	38.12063
Nycteris hispida/aurita	NMK 184937	Kenya	Kakamega	Mungokho Village	0.37485	34.89849
Nycteris hispida/aurita	NMK 184976	Kenya	Kakamega	Mungokho Village	0.37485	34.89849
Nycteris hispida/aurita	MHNG 1971.039	Malawi		Mt Mulanje, Illingworth forest	-16.02775	35.52214
Nycteris hispida/aurita	MHNG 1971.04	Malawi		Mt Mulanje, Illingworth forest	-16.02775	35.52214
Nycteris hispida/aurita	FMNH 225217	Rwanda		Akagera National Park, Bacherie Fishing Camp	-1.89255	30.73215
Nycteris hispida/aurita	FMNH 225218	Rwanda		Akagera National Park, Ruzizi tented camp	-1.90634	30.71696
Nycteris hispida/aurita	FMNH 225240	Rwanda		Lake Gashora	-2.19315	30.23961
Nycteris hispida/aurita	FMNH 195607	South Africa		Ehlanzeni Municipality, Ingwenya Farm (409 SU)	-25.56278	31.85066
Nycteris hispida/aurita	FMNH 151191	Tanzania		Gonja Forest Reserve, 2 km SW Gonja Maore	-4.2667	38.0333
Nycteris hispida/aurita	FMNH 232892	Uganda		Bikongoro Village, Buliisa	2.076738	31.43591
Nycteris hispida/aurita	FMNH 232893	Uganda		Bikongoro Village, Buliisa	2.076738	31.43591
Nycteris hispida/aurita	FMNH 137625	Uganda		Bugala Island, Kalangala, 1.0 km N, 0.5 km E	-0.005278	0.538056
Nycteris hispida/aurita	FMNH 137626	Uganda		Bugala Island, Kalangala, 1.0 km N, 0.5 km E	-0.005278	0.538056
Nycteris hispida/aurita	FMNH 232902	Uganda		Bugungu Wildlife Reserve, Bugungu	1.880648	31.47487
Nycteris hispida/aurita	FMNH 232903	Uganda		Bugungu Wildlife Reserve, Bugungu	1.880648	31.47487
Nycteris hispida/aurita	FMNH 232904	Uganda		Bugungu Wildlife Reserve, Bugungu	1.880648	31.47487

Nycteris hispida/aurita	FMNH 232905	Uganda		Bugungu Wildlife Reserve, Bugungu	1.880648	31.47487
Nycteris hispida/aurita	FMNH 232906	Uganda		Bugungu Wildlife Reserve, Bugungu	1.886645	31.43456
Nycteris hispida/aurita	FMNH 232908	Uganda		Bugungu Wildlife Reserve, Bugungu	1.967507	31.51539
Nycteris hispida/aurita	FMNH 165131	Uganda		Nyabyeya Forestry College, Budongo Forest	1.68333	31.53333
Nycteris javanica	ROM 101970	Indonesia, Borneo		Bukit Soeharto Experimental Forest, 60 km S of Samarinda	-0.5	117
Nycteris macrotis 1	FMNH 216029	Kenya	Malindi	Arabuko-Sokoke Forest, KWS Headquarters	-3.300017	39.99513
Nycteris macrotis 1	FMNH 216033	Kenya	Malindi	Arabuko-Sokoke Forest, KWS Headquarters	-3.300017	39.99513
Nycteris macrotis 1	FMNH 220492	Kenya	Kilifi	Arabuko-Sokoke National Park, Pipit Campsite	-3.29986	39.99509
Nycteris macrotis 1	FMNH 220494	Kenya	Kilifi	Arabuko-Sokoke National Park, Pipit Campsite	-3.29986	39.99509
Nycteris macrotis 1	FMNH 216036	Kenya	Makueni	Chyulu Hills National Park, Campsite	-2.49922	37.93981
Nycteris macrotis 1	FMNH 220981	Kenya	Makueni	Chyulu Hills National Park, Campsite	-2.49922	37.93981
Nycteris macrotis 1	FMNH 216030	Kenya	Malindi	Gedi Ruins	-3.3091	40.01763
Nycteris macrotis 1	FMNH 216031	Kenya	Malindi	Gedi Ruins	-3.3091	40.01763
Nycteris macrotis 1	FMNH 216032	Kenya	Malindi	Gedi Ruins	-3.3091	40.01763
Nycteris macrotis 1	FMNH 216034	Kenya	Malindi	Mango orchard on E side of Malindi-Mombasa Hwy, 300 m N KWS Hdqs	-3.30325	39.99888
Nycteris macrotis 1	FMNH 216035	Kenya	Malindi	Mango orchard on E side of Malindi-Mombasa Hwy, 300 m N KWS Hdqs	-3.30325	39.99888
Nycteris macrotis 1	FMNH 220742	Kenya	Meru	Meru National Park, Kanjoo Gate Compound	0.219817	38.06498
Nycteris macrotis 1	FMNH 220744	Kenya	Meru	Meru National Park, Kanjoo Gate Compound	0.219817	38.06498
Nycteris macrotis 1	FMNH 220745	Kenya	Meru	Meru National Park, Kanjoo Gate Compound	0.219817	38.06498
Nycteris macrotis 1	FMNH 215541	Kenya	Homa Bay	Ruma National Park, houses outside of Headquarters Gate	-0.65174	34.34336
Nycteris macrotis 1	FMNH 220977	Kenya	Taita-Taveta	Tsavo West National Park, Komboyo Campsite	-2.75443	38.11489
Nycteris macrotis 1	FMNH HB61	South Sudan		Badingilo National Park, Bala Pool, Wildlife HQs	5.305333	31.84858
Nycteris macrotis 1	FMNH HB62	South Sudan		Badingilo National Park, Bala Pool, Wildlife HQs	5.305333	31.84858
Nycteris macrotis 1	FMNH HB115	South Sudan		Badingilo National Park, Kweregik	5.30438	31.85166
Nycteris macrotis 1	FMNH HB121	South Sudan		Nimule National Park, Paanzalla	3.585217	31.9834
Nycteris macrotis 1	FMNH HB122	South Sudan		Nimule National Park, Paanzalla	3.585217	31.9834
Nycteris macrotis 1	FMNH HB124	South Sudan		Nimule National Park, Paanzalla	3.585217	31.9834

Nycteris macrotis 1	FMNH 192937	Tanzania		Minziro Forest	-1.071261	31.5479
Nycteris macrotis 1	FMNH 219069	Tanzania		Ruaha National Park, Maji Moto	-8.03658	34.50236
Nycteris macrotis 1	FMNH 219068	Tanzania		Ruaha National Park, Mikindi Springs	-7.75111	34.55189
Nycteris macrotis 1	FMNH 219239	Tanzania		Ruaha National Park, Mikindi Springs	-7.75111	34.55189
Nycteris macrotis 1	FMNH 232911	Uganda		Bugungu Wildlife Reserve, Bugungu	1.829568	31.41269
Nycteris macrotis 1	FMNH 232912	Uganda		Bugungu Wildlife Reserve, Bugungu	1.880648	31.47487
Nycteris macrotis 1	FMNH 232913	Uganda		Bugungu Wildlife Reserve, Bugungu	1.880648	31.47487
Nycteris macrotis 1	FMNH 232914	Uganda		Bugungu Wildlife Reserve, Bugungu	1.967507	31.51539
Nycteris macrotis 1	FMNH 232915	Uganda		Bugungu Wildlife Reserve, Bugungu	1.967507	31.51539
Nycteris macrotis 1	FMNH 232916	Uganda		Bugungu Wildlife Reserve, Bugungu	1.967507	31.51539
Nycteris macrotis 1	FMNH 232917	Uganda		Bugungu Wildlife Reserve, Bugungu	1.967507	31.51539
Nycteris macrotis 1	FMNH 223200	Uganda		Mabira FR, Nagojje station, 0 .79 km NE of Nagojje	0.445128	32.88876
Nycteris macrotis 1	FMNH 223660	Uganda		Murchison Falls NP, 1 km S Ayago R, weir site	2.34000	31.926
Nycteris macrotis 2	FMNH 220739	Kenya	Meru	Meru National Park, Leopard Rock Lodge	0.222566	38.20085
Nycteris macrotis 2	FMNH 220740	Kenya	Meru	Meru National Park, Leopard Rock Lodge	0.222566	38.20085
Nycteris macrotis 3	FMNH 226237	Malawi		Namizimu Forest Reserve, 1.7 km N of Kwitunji Camp	-14.18875	35.38036
Nycteris macrotis 3	FMNH 228897	Mozambique		Chihalatan Caves (Gerhard's Cave), 38.2 km E Inhassoro	-21.671	34.864
Nycteris macrotis 3	FMNH 228898	Mozambique		Chihalatan Caves (Gerhard's Cave), 38.2 km E Inhassoro	-21.671	34.864
Nycteris macrotis 3	FMNH 228899	Mozambique		Chihalatan Caves (Gerhard's Cave), 38.2 km E Inhassoro	-21.671	34.864
Nycteris macrotis 3	FMNH 228900	Mozambique		Chihalatan Caves (Gerhard's Cave), 38.2 km E Inhassoro	-21.671	34.864
Nycteris macrotis 3	FMNH 228901	Mozambique		Chihalatan Caves (Gerhard's Cave), 38.2 km E Inhassoro	-21.671	34.864
Nycteris nana 1	FMNH 227448	Central African Republic		Parc National de Dzanga-Ndoki, Mabea Bai, 21.4 kms NE Bayanga	3.0333	16.41
Nycteris nana 2	FMNH 227446	Central African Republic		Parc National de Dzanga-Ndoki, Mabea Bai, 21.4 kms NE Bayanga	3.0333	16.41
Nycteris nana 2	FMNH 167764	Gabon		Aire d'Exploitation Rationelle de Faune des	-2.227778	10.39444

				Monts Doudou, 25.2 km NW Doussala		
Nycteris thebaica 1	FMNH 225423	Kenya	Narok	Mara Conservancy, Iseiya Public Campsite, adjacent to Serena-Mara Conservancy Headquarters	-1.40065	35.01825
Nycteris thebaica 1	FMNH 225424	Kenya	Narok	Mara Conservancy, Iseiya Public Campsite, adjacent to Serena-Mara Conservancy Headquarters	-1.40065	35.01825
Nycteris thebaica 1	FMNH 225425	Kenya	Narok	Mara Conservancy, Iseiya Public Campsite, adjacent to Serena-Mara Conservancy Headquarters	-1.40065	35.01825
Nycteris thebaica 1	FMNH 225426	Kenya	Narok	Mara Conservancy, Iseiya Public Campsite, adjacent to Serena-Mara Conservancy Headquarters	-1.40065	35.01825
Nycteris thebaica 1	FMNH 225420	Kenya	Narok	Masai Mara National Reserve, Keekorok Lodge	-1.58963	35.23411
Nycteris thebaica 1	FMNH 225421	Kenya	Narok	Masai Mara National Reserve, Keekorok Lodge	-1.58963	35.23411
Nycteris thebaica 1	FMNH 225406	Kenya	Narok	Masai Mara National Reserve, Sarova Mara Lodge	-1.53105	35.32022
Nycteris thebaica 1	FMNH 225407	Kenya	Narok	Masai Mara National Reserve, Sarova Mara Lodge	-1.53105	35.32022
Nycteris thebaica 1	FMNH 225408	Kenya	Narok	Masai Mara National Reserve, Sarova Mara Lodge	-1.53105	35.32022
Nycteris thebaica 1	FMNH 225409	Kenya	Narok	Masai Mara National Reserve, Sarova Mara Lodge	-1.53105	35.32022
Nycteris thebaica 1	FMNH 225410	Kenya	Narok	Masai Mara National Reserve, Sarova Mara Lodge	-1.53105	35.32022
Nycteris thebaica 1	FMNH 225411	Kenya	Narok	Masai Mara National Reserve, Sarova Mara Lodge	-1.53105	35.32022
Nycteris thebaica 1	FMNH 225412	Kenya	Narok	Masai Mara National Reserve, Sarova Mara Lodge	-1.53105	35.32022
Nycteris thebaica 1	FMNH 225413	Kenya	Narok	Masai Mara National Reserve, Sarova Mara Lodge	-1.53105	35.32022
Nycteris thebaica 1	FMNH 225442	Kenya	Narok	Masai Mara National Reserve, Sarova Mara Lodge	-1.53105	35.32022
Nycteris thebaica 1	FMNH 225443	Kenya	Narok	Masai Mara National Reserve, Sarova Mara	-1.53105	35.32022

				Lodge		
Nycteris thebaica 1	FMNH 225444	Kenya	Narok	Masai Mara National Reserve, Sarova Mara Lodge	-1.53105	35.32022
Nycteris thebaica 1	FMNH 225446	Kenya	Narok	Masai Mara National Reserve, Sarova Mara Lodge	-1.53105	35.32022
Nycteris thebaica 1	FMNH 225447	Kenya	Narok	Masai Mara National Reserve, Sarova Mara Lodge	-1.53105	35.32022
Nycteris thebaica 1	FMNH 225448	Kenya	Narok	Masai Mara National Reserve, Sarova Mara Lodge	-1.53105	35.32022
Nycteris thebaica 1	FMNH 225449	Kenya	Narok	Masai Mara National Reserve, Sarova Mara Lodge	-1.53105	35.32022
Nycteris thebaica 1	FMNH 225450	Kenya	Narok	Masai Mara National Reserve, Sarova Mara Lodge	-1.53105	35.32022
Nycteris thebaica 1	FMNH 225451	Kenya	Narok	Masai Mara National Reserve, Sarova Mara Lodge	-1.53105	35.32022
Nycteris thebaica 1	FMNH 225452	Kenya	Narok	Masai Mara National Reserve, Sarova Mara Lodge	-1.53105	35.32022
Nycteris thebaica 1	FMNH 225453	Kenya	Narok	Masai Mara National Reserve, Sarova Mara Lodge	-1.53105	35.32022
Nycteris thebaica 1	FMNH 225454	Kenya	Narok	Masai Mara National Reserve, Sarova Mara Lodge	-1.53105	35.32022
Nycteris thebaica 1	FMNH 225455	Kenya	Narok	Masai Mara National Reserve, Sarova Mara Lodge	-1.53105	35.32022
Nycteris thebaica 1	FMNH 225456	Kenya	Narok	Masai Mara National Reserve, Sarova Mara Lodge	-1.53105	35.32022
Nycteris thebaica 1	FMNH 225457	Kenya	Narok	Masai Mara National Reserve, Sarova Mara Lodge	-1.53105	35.32022
Nycteris thebaica 1	FMNH 225458	Kenya	Narok	Masai Mara National Reserve, Sarova Mara Lodge	-1.53105	35.32022
Nycteris thebaica 1	FMNH 225459	Kenya	Narok	Masai Mara National Reserve, Sarova Mara Lodge	-1.53105	35.32022
Nycteris thebaica 1	FMNH 225460	Kenya	Narok	Masai Mara National Reserve, Sarova Mara Lodge	-1.53105	35.32022
Nycteris thebaica 1	FMNH 225461	Kenya	Narok	Masai Mara National Reserve, Sarova Mara	-1.53105	35.32022

				Lodge		
Nycteris thebaica 1	FMNH 225462	Kenya	Narok	Masai Mara National Reserve, Sarova Mara Lodge	-1.53105	35.32022
Nycteris thebaica 1	FMNH 225463	Kenya	Narok	Masai Mara National Reserve, Sarova Mara Lodge	-1.53105	35.32022
Nycteris thebaica 1	FMNH 225464	Kenya	Narok	Masai Mara National Reserve, Sarova Mara Lodge	-1.53105	35.32022
Nycteris thebaica 1	FMNH 225465	Kenya	Narok	Masai Mara National Reserve, Sarova Mara Lodge	-1.53105	35.32022
Nycteris thebaica 1	FMNH 225466	Kenya	Narok	Masai Mara National Reserve, Sarova Mara Lodge	-1.53105	35.32022
Nycteris thebaica 1	FMNH 225467	Kenya	Narok	Masai Mara National Reserve, Sarova Mara Lodge	-1.53105	35.32022
Nycteris thebaica 1	FMNH 225468	Kenya	Narok	Masai Mara National Reserve, Sarova Mara Lodge	-1.53105	35.32022
Nycteris thebaica 1	FMNH 225469	Kenya	Narok	Masai Mara National Reserve, Sarova Mara Lodge	-1.53105	35.32022
Nycteris thebaica 1	FMNH 225470	Kenya	Narok	Masai Mara National Reserve, Sarova Mara Lodge	-1.53105	35.32022
Nycteris thebaica 1	FMNH 225471	Kenya	Narok	Masai Mara National Reserve, Sarova Mara Lodge	-1.53105	35.32022
Nycteris thebaica 1	FMNH 225472	Kenya	Narok	Masai Mara National Reserve, Sarova Mara Lodge	-1.53105	35.32022
Nycteris thebaica 1	FMNH 225473	Kenya	Narok	Masai Mara National Reserve, Sarova Mara Lodge	-1.53105	35.32022
Nycteris thebaica 1	FMNH 225474	Kenya	Narok	Masai Mara National Reserve, Sarova Mara Lodge	-1.53105	35.32022
Nycteris thebaica 1	FMNH 225241	Rwanda		Kinihira	-2.15592	29.62351
Nycteris thebaica 1	FMNH 225242	Rwanda		Kinihira	-2.15592	29.62351
Nycteris thebaica 1	FMNH 225243	Rwanda		Kinihira	-2.15592	29.62351
Nycteris thebaica 1	FMNH 225244	Rwanda		Kinihira	-2.15592	29.62351
Nycteris thebaica 1	FMNH 225245	Rwanda		Kinihira	-2.15592	29.62351
Nycteris thebaica 1	FMNH 225210	Rwanda		Susa Cave	-1.50575	29.61465

Nycteris thebaica 2	FMNH 198085	Tanzania		Urban Region, West District, Dole, Masingini Forest Station	-6.10155	39.24333
Nycteris thebaica 2	FMNH 198086	Tanzania		Urban Region, West District, Dole, Masingini Forest Station	-6.10155	39.24333
Nycteris thebaica 2	FMNH 198087	Tanzania		Urban Region, West District, Dole, Masingini Forest Station	-6.10155	39.24333
Nycteris thebaica 2	FMNH 198088	Tanzania		Urban Region, West District, Dole, Masingini Forest Station	-6.10155	39.24333
Nycteris thebaica 2	FMNH 198089	Tanzania		Urban Region, West District, Dole, Masingini Forest Station	-6.10155	39.24333
Nycteris thebaica 2	FMNH 147220	Tanzania		West Usambara Mts, 14.5 km NW Korogwe, Ambangulu Tea Estate	-5.05	38.38334
Nycteris thebaica 3	NMK 184759	Kenya	Nakuru	Gilgil, Pipeline Cave	-0.53911	36.29431
Nycteris thebaica 3	NMK 184854	Kenya	Nakuru	Gilgil, Pipeline Cave	-0.53911	36.29431
Nycteris thebaica 3	NMK 184855	Kenya	Nakuru	Gilgil, Pipeline Cave	-0.53911	36.29431
Nycteris thebaica 3	NMK 187337	Kenya	Nakuru	Gilgil, Pipeline Cave	-0.53911	36.29431
Nycteris thebaica 3	NMK 187338	Kenya	Nakuru	Gilgil, Pipeline Cave	-0.53911	36.29431
Nycteris thebaica 3	NMK 187339	Kenya	Nakuru	Gilgil, Pipeline Cave	-0.53911	36.29431
Nycteris thebaica 3	NMK 187340	Kenya	Nakuru	Gilgil, Pipeline Cave	-0.53911	36.29431
Nycteris thebaica 3	NMK 187341	Kenya	Nakuru	Gilgil, Pipeline Cave	-0.53911	36.29431
Nycteris thebaica 3	NMK 187450	Kenya	Kakamega	Kakamega Forest National Reserve, Buyangu KWS HQ	0.355	34.86567
Nycteris thebaica 3	FMNH 215536	Kenya	Kakamega	Kakamega National Reserve, Ikhondo Campground	0.3523	34.86469
Nycteris thebaica 3	FMNH 215537	Kenya	Kakamega	Kakamega National Reserve, Ikhondo Campground	0.3523	34.86469
Nycteris thebaica 3	FMNH 215538	Kenya	Kakamega	Kakamega National Reserve, Ikhondo Campground	0.3523	34.86469
Nycteris thebaica 3	FMNH 215542	Kenya	Nakuru	Lake Nakuru National Park, Lion Hill Cave	-0.3459	36.11916
Nycteris thebaica 3	FMNH 215543	Kenya	Nakuru	Lake Nakuru National Park, Lion Hill Cave	-0.3459	36.11916
Nycteris thebaica 3	FMNH 215544	Kenya	Nakuru	Lake Nakuru National Park, Lion Hill Cave	-0.3459	36.11916
Nycteris thebaica 3	FMNH 215545	Kenya	Nakuru	Lake Nakuru National Park, Lion Hill Cave	-0.3459	36.11916
Nycteris thebaica 3	FMNH 225400	Kenya	Nakuru	Lake Nakuru National Park, Lion Hill Cave	-0.3459	36.11916

Nycteris thebaica 3	FMNH 225401	Kenya	Nakuru	Lake Nakuru National Park, Lion Hill Cave	-0.3459	36.11916
Nycteris thebaica 3	FMNH 225402	Kenya	Nakuru	Lake Nakuru National Park, Lion Hill Cave	-0.3459	36.11916
Nycteris thebaica 3	FMNH 225403	Kenya	Nakuru	Lake Nakuru National Park, Lion Hill Cave	-0.3459	36.11916
Nycteris thebaica 3	FMNH 225404	Kenya	Nakuru	Lake Nakuru National Park, Lion Hill Cave	-0.3459	36.11916
Nycteris thebaica 3	FMNH 225405	Kenya	Nakuru	Lake Nakuru National Park, Lion Hill Cave	-0.3459	36.11916
Nycteris thebaica 3	NMK 184520	Kenya	Laikipia	Loll Daiga Hills Conservancy, Farm House	0.20138	37.12987
Nycteris thebaica 3	NMK 184521	Kenya	Laikipia	Loll Daiga Hills Conservancy, Farm House	0.20138	37.12987
Nycteris thebaica 3	NMK 184522	Kenya	Laikipia	Loll Daiga Hills Conservancy, Farm House	0.20138	37.12987
Nycteris thebaica 3	NMK 184407	Kenya	Laikipia	Loll Daiga Hills Conservancy, Kiburuti Bridge	0.30823	37.15246
Nycteris thebaica 3	NMK 184636	Kenya	Laikipia	Loll Daiga Hills Conservancy,West Kiburuti Borehole Dam	0.18764	37.08247
Nycteris thebaica 3	FMNH 220741	Kenya	Meru	Meru National Park, Leopard Rock Lodge	0.222566	38.20085
Nycteris thebaica 3	NMK 185133	Kenya	Kitui	Mwingi, Nuu, Khaluku Rock Dam	-0.99208	38.33025
Nycteris thebaica 3	NMK 184658	Kenya	Baringo	Ol Jogi Conservancy, Pyramid Camp	0.30933	36.07609
Nycteris thebaica 3	FMNH 232429	Uganda		Agoro Agu Forest Reserve	3.81039	32.92264
Nycteris thebaica 3	FMNH 232430	Uganda		Agoro Agu Forest Reserve	3.81039	32.92264
Nycteris thebaica 3	FMNH 232431	Uganda		Agoro Agu Forest Reserve	3.81039	32.92264
Nycteris thebaica 3	FMNH 232919	Uganda		Bugungu Wildlife Reserve, Bugungu	1.967507	31.51539
Nycteris thebaica 3	FMNH 232109	Uganda		Mt Morungole	3.81083	34.02931
Nycteris thebaica 4	FMNH 216037	Kenya	Malindi	Arabuko-Sokoke Forest, KWS Headquarters	-3.300017	39.99513
Nycteris thebaica 4	FMNH 216039	Kenya	Malindi	Arabuko-Sokoke Forest, KWS Headquarters	-3.300017	39.99513
Nycteris thebaica 4	FMNH 216042	Kenya	Malindi	Arabuko-Sokoke Forest, KWS Headquarters	-3.300017	39.99513
Nycteris thebaica 4	FMNH 216043	Kenya	Malindi	Arabuko-Sokoke Forest, KWS Headquarters	-3.300017	39.99513
Nycteris thebaica 4	FMNH 220447	Kenya	Kilifi	Arabuko-Sokoke National Park, Pipit Campsite	-3.29986	39.99509
Nycteris thebaica 4	FMNH 216040	Kenya	Malindi	Gedi Ruins	-3.3091	40.01763
Nycteris thebaica 4	FMNH 220484	Kenya	Kwale	Kenya Forest Service, Kwale Office, Director's House	-4.173748	39.45206
Nycteris thebaica 4	FMNH 220485	Kenya	Kwale	Kenya Forest Service, Kwale Office, Director's House	-4.173748	39.45206
Nycteris thebaica 4	FMNH 220486	Kenya	Kwale	Kenya Forest Service, Kwale Office, Director's House	-4.173748	39.45206
Nycteris thebaica 4	FMNH 220487	Kenya	Kwale	Kenya Forest Service, Kwale Office, Director's	-4.173748	39.45206

				House		
Nycteris thebaica 4	FMNH 220488	Kenya	Kwale	Kenya Forest Service, Kwale Office, Director's House	-4.173748	39.45206
Nycteris thebaica 4	FMNH 220446	Kenya	Kwale	Shimba Hills National Reserve, Sable Bandas	-4.21521	39.45133
Nycteris thebaica 4	FMNH 220448	Kenya	Kwale	Shimba Hills National Reserve, Sable Bandas	-4.21521	39.45133
Nycteris thebaica 4	FMNH 220449	Kenya	Kwale	Shimba Hills National Reserve, Sable Bandas	-4.21521	39.45133
Nycteris thebaica 4	FMNH 220450	Kenya	Kwale	Shimba Hills National Reserve, Sable Bandas	-4.21521	39.45133
Nycteris thebaica 4	FMNH 220451	Kenya	Kwale	Shimba Hills National Reserve, Sable Bandas	-4.21521	39.45133
Nycteris thebaica 4	FMNH 220452	Kenya	Kwale	Shimba Hills National Reserve, Sable Bandas	-4.21521	39.45133
Nycteris thebaica 4	FMNH 220453	Kenya	Kwale	Shimba Hills National Reserve, Sable Bandas	-4.21521	39.45133
Nycteris thebaica 4	FMNH 220455	Kenya	Kwale	Shimba Hills National Reserve, Sable Bandas	-4.21521	39.45133
Nycteris thebaica 5	FMNH 213626	Mozambique		Ilha de Mozambique, Museu da Ilha de Mozambique	-15.03513	40.73499
Nycteris thebaica 5	FMNH 213627	Mozambique		Ilha de Mozambique, Museu da Ilha de Mozambique	-15.03513	40.73499
Nycteris thebaica 5	FMNH 213628	Mozambique		Ilha de Mozambique, Museu da Ilha de Mozambique	-15.03513	40.73499
Nycteris thebaica 5	FMNH 213629	Mozambique		Ilha de Mozambique, Museu da Ilha de Mozambique	-15.03513	40.73499
Nycteris thebaica 5	FMNH 213630	Mozambique		Ilha de Mozambique, Museu da Ilha de Mozambique	-15.03513	40.73499
Nycteris thebaica 5	FMNH 213631	Mozambique		Ilha de Mozambique, Museu da Ilha de Mozambique	-15.03513	40.73499
Nycteris thebaica 5	FMNH 213632	Mozambique		Kissona village	-15.06079	40.66266
Nycteris thebaica 6	FMNH 226240	Malawi		Mangochi Hills, above Skull Rock Scout Camp	-14.51389	35.46478
Nycteris thebaica 6	FMNH 226238	Malawi		Namizimu Forest Reserve, 1.7 km N of Kwitunji Camp	-14.18875	35.38036
Nycteris thebaica 6	FMNH 187360	Tanzania		Juani Island	-7.99285	39.79242
Nycteris thebaica 6	FMNH 187412	Tanzania		Juani Island	-7.99285	39.79242
Nycteris thebaica 6	FMNH 193210	Tanzania		Kibebe Farms, approx 6 km ESE Iringa	-7.7978	35.7567
Nycteris thebaica 6	FMNH 187361	Tanzania		Mafia Island, Utende	-7.9761	39.74447
Nycteris thebaica 6	FMNH 219066	Tanzania		Ruaha National Park, Mikindi Springs	-7.75111	34.55189

Nycteris thebaica 6	FMNH 219067	Tanzania		Ruaha National Park, Mikindi Springs	-7.75111	34.55189
Nycteris tragata	LSUMZ 4413	Malaysia				
Nycteris tragata	TTU 108180	Malaysia		Krau Game Reserve	3.596	102.1817
Coleura afra	FMNH 220403	Kenya	Kwale	Fikirini, Three Sisters, Mbenyenye Cave		

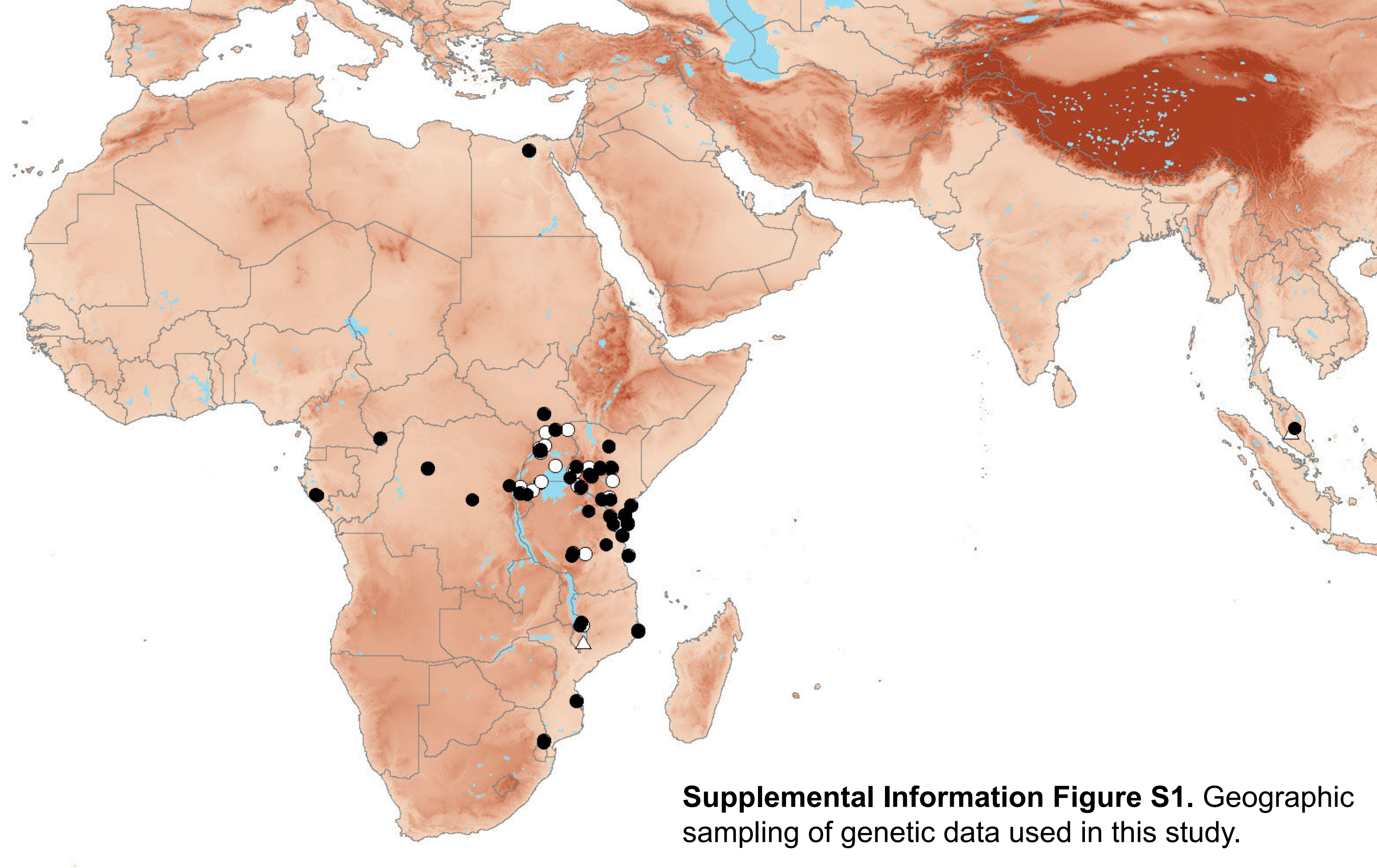

Supplemental Information Figure S1. Geographic sampling of genetic data used in this study.

Supplemental Information Figure S2. Species tree inferred in StarBEAST for *Nycteris* for 21 clades, including *Nycteris thebaica* clades 1 to 6.

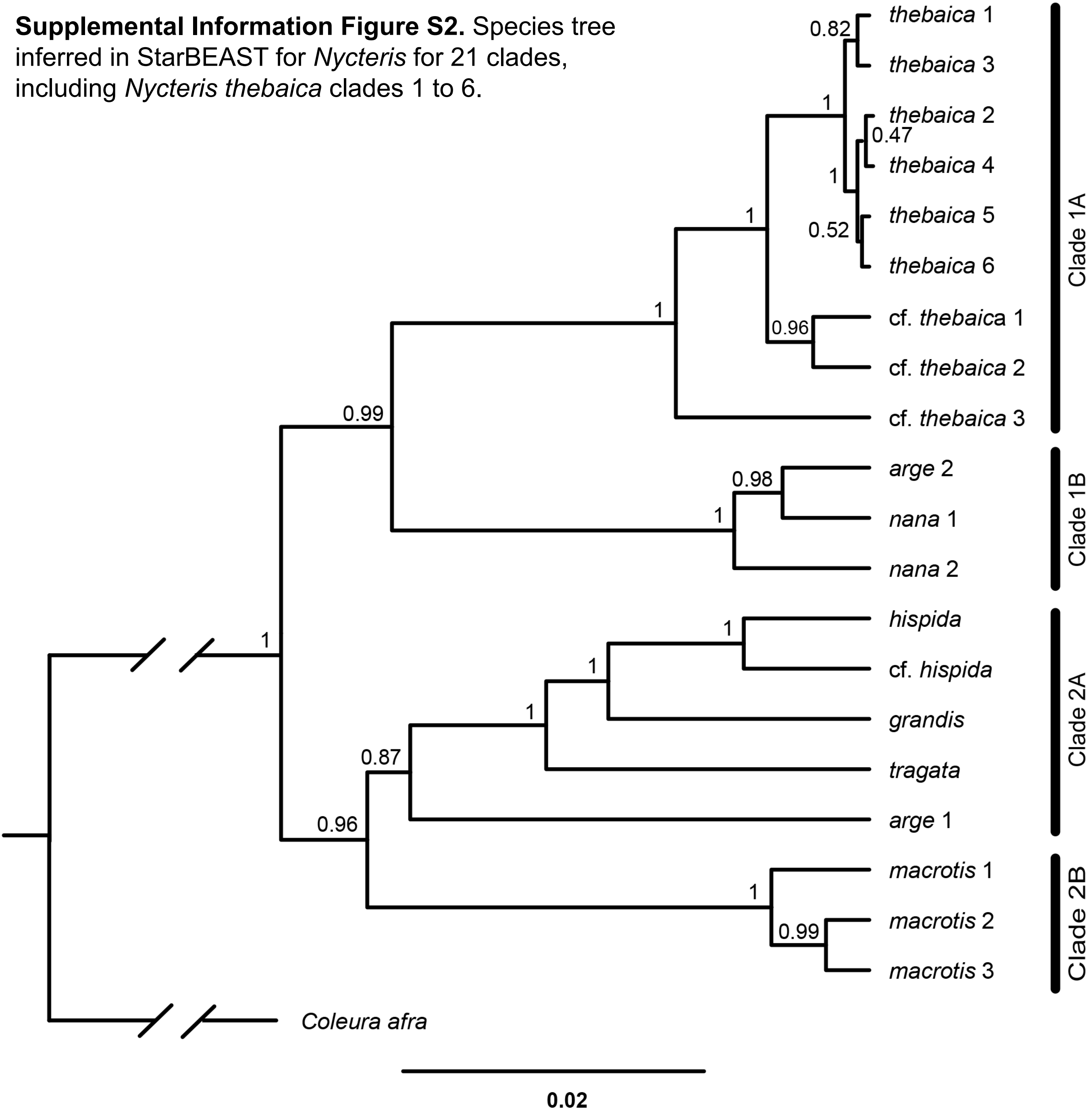

Supplement: Supplementary file 1 — Table S1. List of locality data for specimens used in genetic analyses of Nycteris. Figure S1. Geographic sampling of genetic data used in this study. Plotting symbols denote the locations of one or more individuals represented by mitochondrial sequence (cytb) downloaded from GenBank (+), those represented only by cytb data newly generated for this study (open circles), and those where both mitochondrial and nuclear sequences were newly generated (filled circles). Taxon, localities, and coordinates for these points are included in Supporting Information Table S1. Figure S2. Species tree inferred in StarBEAST for Nycteris for 21 clades, including Nycteris thebaica clades 1 to 6. Nodes are labeled with posterior probabilities. [file JZS-57-1019-s001.pdf]
